# Stochastic adaptive-service level agreement-based energy management model for smart grid and prosumers

**DOI:** 10.1371/journal.pone.0278324

**Published:** 2022-12-13

**Authors:** Waqar Ahmed, Bilal Khan, Zahid Ullah, Faizan Mehmood, Sahibzada Muhammad Ali, Ernest Edem Edifor, Sajid Siraj, Raheel Nawaz

**Affiliations:** 1 Department of Electrical and Computer Engineering, CUI Abbottabad Campus, Abbottabad, Pakistan; 2 Department of Electrical Engineering, UMT Lahore, Sialkot Campus, Sialkot, Pakistan; 3 Department of Electrical Engineering, University of Engineering and Technology, Taxila, Pakistan; 4 Manchester Metropolitan University, Manchester, United Kingdom; 5 University of Leeds, Leeds, United Kingdom; 6 Staffordshire University, Stoke-on-Trent, United Kingdom; University of Science and Technology of China, CHINA

## Abstract

The growing issue of demand-supply management between the prosumers and the local energy market requires an efficient and reliable energy management model. The microlayers, such as prosumers, energy districts, and macro players, namely retail dealers and wholesale dealers play a pivotal role in achieving mutual benefits. The stochastic nature of renewable energy generation in energy districts requires an effective model that can contemplate all stochastic complexities. Therefore, this paper proposes a mutual trade model between energy districts and smart grid to authorize the prosumers for mutual energy transactions under the stochastic adaptive-service level agreement. Moreover, multiple smart contacts are developed between the stakeholders to design adaptability and stochastic behavior of wind speed and solar irradiance. The real-time adaptations of the stochastic adaptive-service level agreement are based on technical beneficial feasibility and achieved through stochastic and adaptive functions. The optimized solution based on a genetic algorithm is proposed for the energy cost and energy surplus of prosumers and output parameters of the mutual trade model (grid revenue). In the context of mutual benefits associated with balanced demand and supply, the economic load dispatch and simplex method maximization are used for optimized demand-supply energy management. Moreover, the effectiveness of the proposed adaptive and stochastic mutual trade model is validated through simulation and statistical analysis.

## 1. Introduction

The energy demand is growing exponentially while fossil fuels are becoming unpopular, and therefore, there is a need to increase the penetration of renewable energy resources in the existing power network along with a better and smarter energy management solution. The International Renewable Energy Agency reported that both government and public partnerships are targeting to reduce the capital cost to meet the total 36% of energy demand from renewable energy sources by 2030 [1]. To overcome the gap between energy supply and demand, energy consumers in many countries are allowed to generate their energy and feed their surplus back into the grid. These types of consumers are usually referred to as “prosumers”. This mutual incentive-based measure allows the energy providers to provide cost-effective, environment-friendly, and sustainable energy solutions. The prosumers are active micro-mini players with small-scale renewable energy generation who participate in the smart grid environment stochastically (as their generation capacity depends on stochasticity) [2].

Although extensive work has been carried out for the development of a prosumer-based smart grid for a single and cluster-based prosumers operation, where each prosumer act as a shareholder of the energy market. A lot of remedies and challenges regarding energy estimation, socio-technic issues, efficient strategies for energy management, and incentive-based programs are still overlooked in the literature. Further, the integration and control of small-scale prosumer-based microgrids in the paradigm of the Internet of Things and demand-side management is a crucial challenge for the future smart grid [3]. The challenge of secure energy trading for blockchain-based peer-to-peer microgrids is addressed in [4–6]. Smart contracts ensure beneficial mutual energy transactions between the smart grid and prosumers.

In the literature, several priority-based energy scheduling mechanisms for a distributed network are proposed. In [7, 8], the active energy transactions for multiple prosumers are prioritized. Although this approach is effective, it does not consider the reliability and congestion aspects. This problem is solved by [9, 10] using transactive energy paradigms and taking distributed renewable energy generation in a local distribution area, respectively. The proposed solutions help to exchange energy between various prosumers and empower distribution system operators to participate in the wholesale electricity market to enhance their energy sharing in the local energy trade market. The layer-wise energy management model with monitoring, smart metering facility, and fast communication links between stakeholders is presented, which gives an advantage in forecasting the electricity demand growth but lacks in providing the randomness and stochasticity of source parameters of renewable energy generation. Service-Level-Agreement (SLA) between the stakeholders requires clear and transparent energy transactions for bidirectional energy and information flow along with pricing schemes [11, 12]. However, the proposed solution is unable to design multiple smart contract-based SLAs with multiple Regions of Convergence (RoC) and Regions of Divergence (RoD) in response to stochastic source parameters. The households belonging to prosumers and consumers were considered in [13], analyzing the energy efficiency of prosumer households. The concept of a community microgrid was introduced using multiple deterministic planning designs, which reduced the investment cost compared to household microgrids [14].

The energy transactions between stakeholders to minimize the mismatch of demand-supply between prosumers are highly effective than traditional cost function minimization [15, 16]. The performance of day-ahead planning and real-time operation supported by the energy storage system is analyzed in [17]. The robust two-stage energy management and sharing model is presented in [18]. The first stage solved the problem of energy sharing schedule and price uncertainties by converting bi-level optimization into mixed-integer linear programming. The second stage is applying an online mechanism for scheduling and imposing penalties on deviated prosumers, who try to adjust their preceding schedule [18].

A sharing economy system using the Internet of Things is presented in [19], which can be deployed when the prosumers are bound to communicate in a binary way and requires less computational, connectivity, and actuation burden on the infrastructure. The virtual association’s concept of the aggregator is presented in [20], in which the fair sharing and min-max optimization techniques are used to prioritize the virtual association and increase competitiveness, and prosumer participation. The mutual interactions between smart grid and renewable energy districts using demand response programs were discussed and comprehensively reviewed [21, 22] to ensure the participation of distributed end-users. A scenario-based algorithm is used for dealing with the intermittency of wind and solar energy in a combined power system resulting in reduced system cost and minimized environmental pollution [23]. The concept of energy coalition using cooperative game theory in which prosumers operation is enhanced by reducing the overall coalition cost through collaboration between the owners of the energy storage system is presented [24]. Further, the Game theory is organized to reward the outperforming players.

The above-mentioned techniques provide a cost-effective solution for mutual energy trading between the Energy Districts (eDs) and smart grids and enhance the prosumer contribution to developing prosumer-based microgrids into modern, controllable, self-healing, and resilient smart grids. However, these works lack in providing (i) multiple Smart Contracts (SCs) based Stochastic and Adaptive SLA (SA-SLA) with multiple RoC and RoD and (ii) demand-supply optimized management using the simplex method and Economic Load Dispatch (ELD).

The key contributions of this paper are given as:

- The development and analysis of a prosumer-based energy management model for mutual energy trading between the eDs (wind and solar) and smart grid. The mutual energy trading between multiple eDs increases energy sharing to reduce the peak energy demand.
- The development of a new SCs-based SA-SLA technique with multiple convergences and divergence regions. The new SA-SLA technique can incorporate prosumer energy generation based on stochastic source parameters, such as solar irradiance and wind speed. The stochasticity of source parameters creates multiple ranges of prosumer energy surplus.
- The validation of prosumer energy cost and grid revenue using Genetic Algorithm, when multiple eDs are interfaced with the smart grid considering Real-Time Pricing (RTP) and Day-ahead (DaH) pricing.
- The development and validation of SA-SLA employing multiple SCs-based smart SLAs. Further, the demand-supply optimized management is achieved using the Simplex method and ELD.

The organization of the paper is as follows. Section II discusses the renewable eDs mutual trade model incorporating environmental shifts and DRPs. Section III presents the layers and attributes of the SA-SLA. A robust optimization model for PES, prosumer energy cost, and grid revenue are discussed in Section IV. The performance evaluation including data analysis, seasonal variations, smart SLAs with multiple RoC, and RoD are provided in Section V. Section VI concludes the paper with major findings of work.

## 2. Renewable energy districts based Mutual Trade Model (MTM)

### 2.1 System model

The SA-SLA-based MTM between renewable energy generation-based eDs (wind and solar) and utility is presented in this section. MTM is a stochastic adaptive model with a bidirectional energy management capability to incorporate various price-based incentives like RTP and DaH pricing. MTM provides an effect on the energy consumption pattern of prosumers considering stochastic weather parameters including dry bulb temperature, dew point, wind speed, and cloud cover. The development of smart grid technologies focuses on introducing a mechanism that can automatically balance energy demand and energy supply by varying desired set points. In this regard, an effective solution to the prominent issue of balancing energy demand supply is presented [25] using a simplex method and ELD. Price-based incentivization of prosumers and demand-side management are key factors to overcome the issue. The demand-side management ensures pertinent benefits like (i) reducing peak hour demand, (b) consumer energy cost reduction, (c) load profile improvement, and (d) best utilization of renewable energy sources [26]. Keeping in view the stochasticity of source parameters, prosumer interrelation, and community-based relations, Stochastic Adaptive Functions (SAFs) of prosumer-generated energy are included in the MTM to model and design the stochastic behavior of eDs under SA-SLA [19].

**Fig 1:**
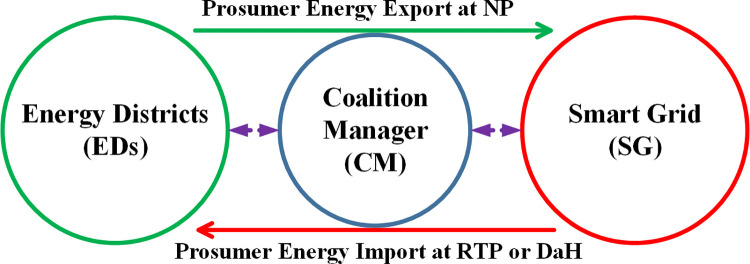
Bidirectional energy flow via CM, NP: Nominal price.

The aggregator plays an important role in enhancing the positive behavior of players [20]. A central control party called Coalition Manager (CM) that regulates the behavior of stakeholders is included in MTM. The role of CM is depicted in Fig 1. SCs are developed between stakeholders under the authority of CM. CM is responsible for updating stakeholders on the upcoming weather conditions, energy demands, energy export, energy import, and electricity tariff of energy import and export (nominal, DaH, or RTP). The CM rewards outperforming stakeholders using game theory [24]. Further, CM is responsible to incentivize outperforming players and penalize stakeholders for violating SA-SLA. Some special energy transactions in the form of import or export offered by the service provider are also communicated to prosumers via CM with details of incentives. Prosumer energy import and export are presented in Fig 1.

### 2.2 Environmental shifts

Environmental dynamics vary quantitatively throughout the year. A reasonable climate variation occurred around the year that changed the energy demand of buildings [27]. Environmental shifts pose a pertinent impact on the energy consumption of prosumers. This paper presents a season-wise analysis of energy consumption as the performance of renewable energy generation (PV arrays and wind turbines), is affected by weather parametric variations and climatic drifts. The energy efficiency of renewable energy generation can be increased using proper weather forecasting techniques [28]. Spring, summer, and winter seasons are considered in this paper to analyze the prosumer energy cost (PEC), prosumer energy surplus (PES), and grid revenue (GR).

### 2.3 Demand response programs (DRPs)

DRPs are playing a vital role in the energy management system of the smart grid [22]. The operational costs of microgrids with distributed renewable energy sources can be reduced to a large extent by the participation of DRPs. DRPs can be categorized as time-based or different incentive-based. In DRPs, end users are encouraged to reduce their energy demands during peak hours and accordingly receive a reward based on the types of DRPs. In this paper, the Price based DRPs (i.e., RTP and DaH pricing schemes) are considered and a comparative analysis is provided. CM playing the role of aggregator decides a pricing scheme and communicates to the stakeholders accordingly [29].

## 3 Stochastic adaptive- service level agreement

**Fig 2:**
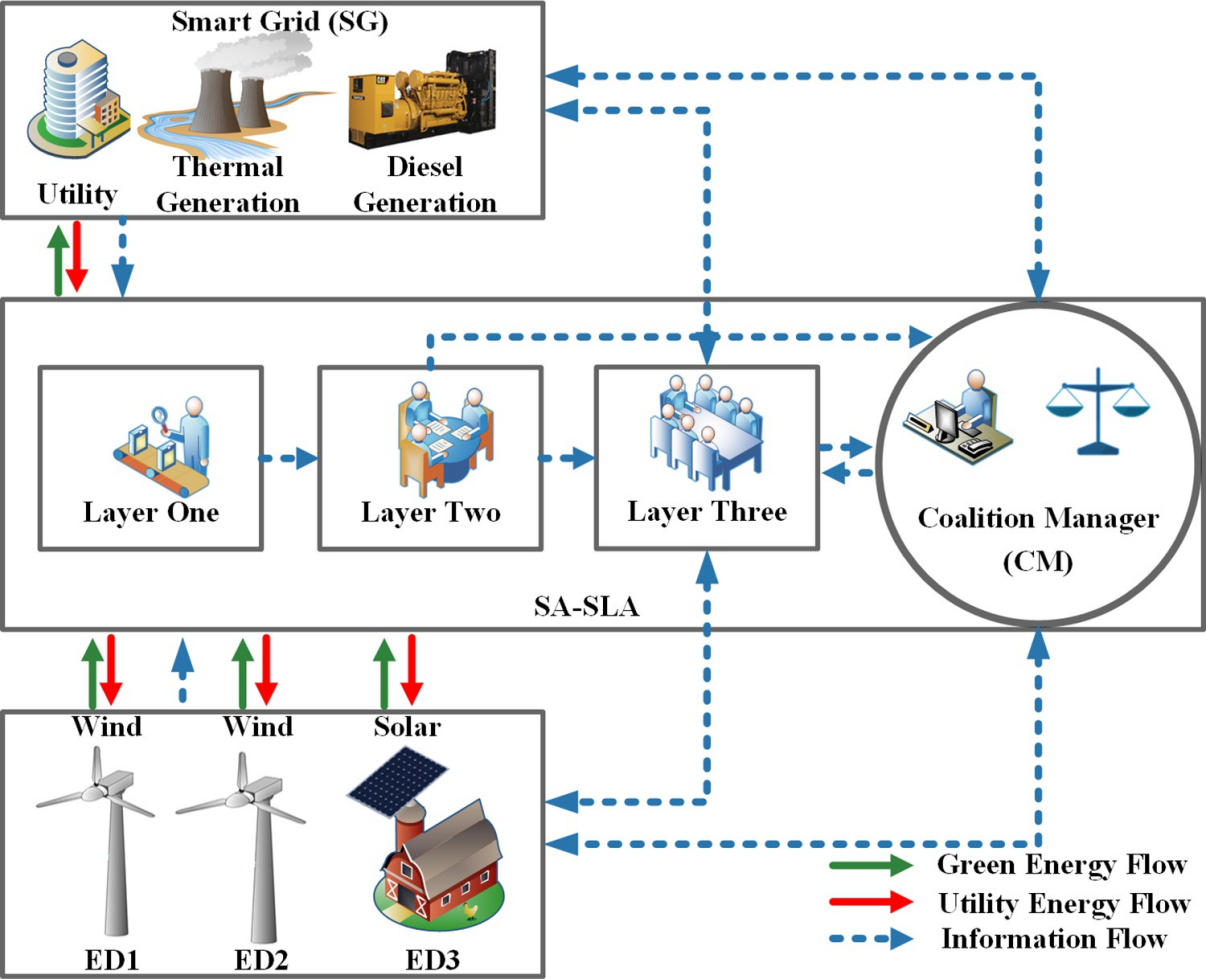
Bidirectional energy flow between energy districts and utility.

SLA is a formal contract between the service provider (smart grid) and prosumers using predefined service standards. The service responsibility, service quality, service reliability, service response time, performance, and security monitoring aspects are mainly covered in SLA. SLA with a new paradigm of active services, transform passive consumers into technology-enabled active players performing as prosumers. Multiple SCs based on the stochastic nature of RES are developed between stakeholders. Utility and eDs associated with adaptations of SA-SLA play a stakeholder role. Maintaining a balance between energy supply and energy demand is necessary to get socio-economic benefits [2]. The proposed SA-SLA with bi-directional communication, real-time monitoring, service security, and multiple contracts assures a balance between energy demand and energy supply. CM regulates the actions of stakeholders by enforcing their energy activities under SA-SLA by specifying typical rewards and penalties as mentioned in (6). The typical block diagram of SA-SLA is shown in Fig 2. Layers play an important role in appropriately regulating the performance of emergent distribution links [2]. The detail of the three layers used in SA-SLA is as follows. Layer one with active agents, players, and vendors consists of multiple SCs, logic, a set of rules, constraints, and service descriptions. The expectations and responsibilities of stakeholders are also defined in layer one. The stakeholders use layer one in the first phase to communicate with the subsequent layers. Considering various SCs and rules, layer one converts stakeholder’s information into a readable form and sends the gathered information to layer two for further processing. Layer two uses directions from CM.

The function of layer two, following the CM direction, is to make decisions based on information prearranged by layer one. A set of If-then rules are involved to evaluate information. After sorting information, layer two, by using if-else commands check the validity of information and sends the decision to both CM and layer three. Layer three is the most trusted and important layer of SA-SLA because CM and stakeholders are directly linked using bidirectional communication. Service security, response time, energy management, and performance of stakeholders are key parameters of layer three. The function of layer three, having instant information of stakeholders, is to send requests of stakeholders to CM for final approval and circulate CM decision for kind information of stakeholders.

The key characteristics of SA-SLA are described as:

**Stochastic Adaptive Functions (SAFs):** Wind and solar energies depend on stochastic environmental parameters (wind speed and solar irradiance) and exhibit highly random behavior. Thus, to capture these energies, it is vital to represent them with some type of functions that approximately follow the same pattern as they do. The Gaussian distribution function is incorporated to capture the energy generated by a wind turbine (WT) and PV arrays.

**Smart Contracts (SCs):** The contract in which energy import and export prices are independent of PES is known as fix contract. Whatever the PES is, a fixed contract reflects no influence on energy import and export prices and gives rise to a fixed SLA. SCs are dynamic contracts linked with PES. SCs define three different ranges of PES, such as PES from 10%-30%, 31%-60%, and 61%-100%. Contracts are smart in the sense that different PES ranges develop multiple SCs, for example, *SC*_1_, *SC*_2_, or *SC*_3_. SCs incorporating different PES ranges are defined as:

$$SCs=\left\{ \begin{array}{l} {SC}_{1}\;for\;10\%\leq E_{S}\leq 30\%\\ {SC}_{2}\;for\;31\%\leq E_{S}\leq 60\%\\ {SC}_{3}\;for\;61\%\leq E_{S}\leq 100\%. \end{array}\right.$$ (1)

where *E*_*S*_ is prosumer energy surplus, while *SC*_1_, *SC*_2_, *and SC*_3_ are smart contracts 1, 2, and 3 respectively.

**Adaptations:** Parametric variations, stochastic patterns, SAFs, domains, ranges, and a set of rules are defined for energy balance adaptations. Stochastic and adaptive environmental parameters with stochastic and adaptive domains and ranges of SAFs stochastically change PES and create mismatches between prosumer energy supply and energy demand (of prosumers or utility). The SA-SLA develops a set of SCs for stochastic energy supply conditions. SCs with different ranges of PES are associated with different prosumer energy import and export prices and result in different PEC with less PEC for more PES. In this manner, each SC creates a specific RoC and RoD for PES and according to PEC.

**Prosumer Energy Demand:** Prosumer energy demand is associated with environmental drifts that greatly influence the energy demand of consumers [12]. The proposed SA-SLA incorporating SAFs adapt these variations caused by stochastic environmental parameters. So, the energy demand of prosumer varies, and variation of stochastic environmental parameters is dealt with SAFs of SA-SLA.

**Utility Loading:** Grid is considered to export energy to or import energy from eDs prosumers. Utility loading conditions could be lightly loaded, mediumly loaded, heavily loaded, or in an emergency condition.

**Convergent and Divergent Contractual Limits:** Depending upon the stochasticity of wind speed and solar irradiance parameters, the SAFs result in different ranges of prosumers generated energy. Multiple ranges of prosumers generated energy results in different PES ranges and assisted in the development of SCs. As Gaussian distribution is considered to present the random behavior of prosumer-generated energy, the area under the probability density curves of SAFs is taken as a region of interest in our proposed SA-SLA. In the outer 5% area, the Probability Density Function (PDF) possesses a very small value in the upper portion while the available energy is too little to meet the energy demand of the prosumer in the lower portion. So, for the proposed SA-SLA, the middle 95% area under the PDF curve is considered RoC while RoD refers to the outer 5% area of SA-SLA.

***Prosumer Mode*:** Whenever the Energy produced by an ED is more than the energy demand, the ED will be able to export the extra energy to Utility under the supervision of CM and this surplus energy is referred to as PES.


$$(Energy\;Surplus)\Rightarrow ({\left(E_{eDs}\right)}_{generation}>{\left(E_{eDs}\right)}_{demand}).$$ (2)

The dependence of PES on Gaussian distributed prosumers generated energy distributes PES in a gaussian manner whenever the ED is in Prosumer mode of operation.

***Consumer Mode*:** Whenever the energy produced by ED is not enough to meet their load, the ED will import energy from utility to meet energy demand and known as PEC.


$$\begin{array}{c} (Energy\;Cost)\Rightarrow \\ ({\left(E_{eDs}\right)}_{generation}<{\left(E_{eDs}\right)}_{demand}). \end{array}$$ (3)

In the consumer mode of operation, the increase in energy generation of ED will decrease energy costs and vice versa. PEC follows inverse relation to ED-generated energy and different PEC will result in multiple SLAs between stakeholders. The smart grid export energy to ED on RTP or DaH pricing.

**Utility Revenue:** The involvement of a prosumer in the smart grid provides benefits to both the prosumer and the smart grid. The benefits-to-cost ratio is the key parameter to determine the utility benefits with corresponding investment. The energy is directly exported by the grid to various consumers and the energy first imported from eDs is at a nominal rate and then exported to the consumer at RTP or DaH price to generate GR.


$$GR=E_{u}\times P_{RTP}+[-{\left(E_{eD}\right)}_{s}\times P_{n}+{\left(E_{eD}\right)}_{s}\times P_{RTP}].$$ (4)

$$\frac{Benefits}{Cost}=\frac{{\left(E_{ED}\right)}_{s,j}\times P_{n}}{{\left(E_{ED}\right)}_{s,j}{\times P}_{RTP}}.$$ (5)

**Utility Cost:** Utility pays prosumers at a nominal price rate for how much energy they export to the utility. So, prosumer energy export to the utility will be considered as a utility cost.

**Utility Benefit:** Exported energy to consumers at RTP or DaH price. Prosumers pay the utility at RTP and DaH price for how much energy they import from the utility. So, prosumer energy import from the utility will be considered as a utility benefit.

**Maximizing Surplus:** Considering the different costs of each ED, the simplex optimization method is used to maximize the surplus with decreased cost of eDs.

***Utility Energy Demand Management*:** demand-supply optimized management optimally utilizes energy sources and increases the GR. To satisfy utility energy demand, the simplex method and ELD are proposed in SA-SLA.

**Rewards and Penalties:** The rewards are associated with the beneficial action and fulfillment of the contract and violation of commitments lead to penalizing the violating body (stakeholder). Amazon EC2 criterion-based rewards and penalties are considered. 10% of the contracted amount will be paid by the penalized body for delay of less than or equal to 1% of total execution time. For the execution time delay of more than 1%, the penalty increases from 10% to 30% of the contracted amount.


$$Penalty=\left\{ \begin{array}{c} ETD\leq 1\%,\;10\%\;of\;\$\\ ETD>1\%,\;30\%\;of\;\$ \end{array}\}.\right.$$ (6)

## 4 Robust Optimization Model (ROM)

SAFs play an important role in ROM to model the variation encountered by highly stochastic wind speed and solar irradiance. The ROM uses SAFs to provide the best possible results based on stochastic source parameters. Stochastic source parameters result in different PES and PEC ranges. Susceptible convergence and divergence limits of SLA are prone to stochastic ranges of PEC and PES. SA-SLA adapts discrepancies of PES and PEC by developing multiple SCs and introduces new beneficial SLA in terms of convergent and divergent limits.

### 4.1 Gaussian distribution functions

Environmental parameters (solar irradiance and wind speed) are stochastic. SA-SLA incorporates SAFs of prosumer-generated energy in response to stochastic parameters (solar irradiance and wind speed). Based on these stochastic parameters, the output energy from WT and PV array approximately follows the Gaussian distribution [30]. The Gaussian distribution function of energy generated is defined as follows:

$$f_{E}(e)=ae^{-\frac{{\left(E-\mu_{E}\right)}^{2}}{2\sigma_{E}^{2}}}.$$ (7)

where $a=\frac{1}{\sqrt{2\pi \sigma_{E}^{2}}}$ represents mean variable length of generated energy, *σ*_*E*_ represents the standard deviation of generated energy, and *μ*_*E*_ represents the mean value of generated energy. In probability theory and statistics, a collection of random variables is Independent and Identically Distributed (i.i.d) if they are mutually independent and each random variable in a collection follows the same probability distribution as its counterparts. As prosumer energy solely depends on independently stochastic source parameters, the random variables associated with prosumer generated energy have i.i.d values. Random vector associated with prosumer generated energy is expressed as follows:

$$E=(E_{t_{1},}E_{t_{2},}\ldots ,E_{t_{k}}).$$ (8)

where $E_{t_{1},}E_{t_{2},}\ldots ,E_{t_{k}}$ are gaussian i.i.d values of prosumer generated energy in the time interval *t*_1_, *t*_2_…,*t*_*k*_. Random variables of random vector exhibit i.i.d values. As each component of the random vector exhibits univariate gaussian distribution, the random vector itself exhibits a multivariate gaussian distribution with k-dimension. A linear combination of random vector components is written as:

$$Y=({d_{1}E}_{t_{1}+}\ldots ,+d_{k}E_{t_{k}}).$$ (9)

For random variable *Y* = *d*^*T*^*Z* and any constant vector *d*∈ℝ^*k*^, random vector follows the multivariate gaussian distribution. The generalized expressions for output power and energy from PV arrays and WT are given in the following subsections.

### 4.2 Stochastic wind models

The ED1 and ED2 are wind energy districts. The output power and energy generated by WT are defined as:

$$G_{w}=\left\{ \begin{array}{l} 0\;v\leq v_{i},v\geq v_{0}\\ \;\frac{v-v_{i}}{v_{r}-v_{i}}G_{w_{r}}\;v_{i}\leq v\leq v_{r}\\ G_{w_{r}}\;v_{r}\leq v\leq v_{o} \end{array}\right.$$ (10)

$$E_{w}=T_{w}\times G_{w}=T_{w}\times \left\{ \begin{array}{l} 0\;v\leq v_{i},v\geq v_{0}\\ \;\frac{v-v_{i}}{v_{r}-v_{i}}G_{w_{r}}\;v_{r}\geq v\geq v_{i}\\ \;G_{w_{r}}\;v_{r}\leq v\leq v_{o} \end{array}\right.$$ (11)

where *G*_*w*_ and $G_{w_{r}}$ represent the actual output power and rated output power of WT respectively, *v* denotes the actual wind speed, *v*_*i*_ denotes the cut-in wind speed, *v*_0_ denotes the cut-out wind speed, *v*_*r*_ denotes the range of rated wind speed, $T_{w}$ denotes the time of operation of WT, and *E*_*w*_ denotes the actual energy generated by WT in time $T_{w}$.

### 4.3 Stochastic solar models

The ED3 is solar ED. Solar PV arrays are employed to generate electrical power. The output power and energy from the PV array, which is stochastic due to varying solar irradiance [31] is defined as:

$$P_{act}=A\times G\times PR\times r.$$ (12)

$$E_{PV}=T_{PV}\times P_{act}=T_{PV}\times A\times G\times PR\times r.$$ (13)

Where G is the annual averaged solar irradiance received on a tilted axis (in *kW*/*m*^2^), PR represents the performance ratio of the PV module, r denotes the solar array yields (in %), A is the PV array’s total area, *P*_*act*_ represents the actual output power of the PV module, $T_{PV}$ represents the time of operation of the PV array and *E*_*PV*_ represents the actual output energy of the PV array.

### 4.4 Prosumer energy surplus

The prosumer energy generation and energy demand fluctuations greatly influenced the PEC, GR, and PES of the smart grid. PES refers to the extra amount of energy that exceeded the energy demand of the prosumer. The bidirectional energy flow between smart grids and prosumers enables prosumers to export the extra energy to utility with multiple SCs under the SA-SLA framework. The PES is maximized while minimizing the PEC. The optimization problem formulation of PES of *j*^*th*^ prosumer is expressed as:

$$max\sum_{h=1}^{H}\left({\left(E_{ED}^{h}\right)}_{gen,j}-{\left(E_{ED}^{h}\right)}_{dem,j}\right).$$ (14)

s.t.


$$0\leq {\left(E_{ED}^{h}\right)}_{gen,j}\leq {\left(E_{ED,r}\right)}_{gen,j},$$ (15)

$$0\leq {\left(E_{ED}^{h}\right)}_{dem,j}\leq {\left(E_{ED,max}\right)}_{dem,j},$$ (16)

$$0\leq {\left(E_{ED}\right)}_{s,j}\leq max(0,{\left(E_{ED}^{h}\right)}_{gen,j}-{\left(E_{ED}^{h}\right)}_{dem,j}).$$ (17)

PES of prosumers is in the range of minimum energy surplus to maximum energy surplus defined as:

$${\left(E_{ED}\right)}_{s,j}=\left\{ \begin{array}{c} {\left(E_{ED,min}\right)}_{s,j}\;for\;({\left(E_{ED,max}\right)}_{dem,j}\&{\left(E_{ED,min}^{h}\right)}_{gen,j})\\ {\left(E_{ED,mean}\right)}_{s,j}\;for\;({\left(E_{ED,mean}\right)}_{dem,j}\&{\left(E_{ED,mean}^{h}\right)}_{gen,j})\\ {\left(E_{ED,max}\right)}_{s,j}\;for\;({\left(E_{ED,min}\right)}_{dem,j}\&{\left(E_{ED,max}^{h}\right)}_{gen,j}). \end{array}\right.$$ (18)

where ${\left(E_{ED}^{h}\right)}_{gen,j}$ represents the actual hourly energy generation of *j*^*th*^ ED, ${\left(E_{ED}^{h}\right)}_{dem,j}$ denotes the actual hourly energy demand of *j*^*th*^ ED, (*E*_*ED*,*max*_)_*dem*,*j*_, (*E*_*ED*,*mean*_)_*dem*,*j*_ and (*E*_*ED*,*min*_)_*dem*,*j*_ represents the maximum, mean, and minimum energy demand of *j*^*th*^ ED respectively, (*E*_*ED*,*r*_)_*gen*,*j*_, (*E*_*ED*,*max*_)_*gen*,*j*_, (*E*_*ED*,*mean*_)_*gen*,*j*_ and (*E*_*ED*,*min*_)_*gen*,*j*_ represents the rated, maximum, mean, and minimum energy generation of *j*^*th*^ ED respectively, (*E*_*ED*_)_*s*,*j*_ is the surplus energy of *j*^*th*^ ED, and (*E*_*ED*,*min*_)_*s*,*j*_, (*E*_*ED*,*mean*_)_*s*,*j*_ and (*E*_*ED*,*max*_)_*s*,*j*_ are the minimum, mean, and maximum energy surplus of *j*^*th*^ ED.

### 4.5 Prosumer energy cost

The energy generated by the prosumer is usually less than the prosumer energy demand because of the stochastic nature of source parameters. Whenever the prosumer energy generation is not enough to meet the prosumer energy demand, additional energy is imported from the utility that incurs a cost on the prosumer called PEC. Prosumer utilizes utility energy based on RTP or DaH pricing schemes as updated by CM. The optimization problem formulation of PEC of *j*^*th*^ prosumer is expressed as:

$$min\sum_{h=1}^{H}P_{RTP}^{h}\times ({\left(E_{EDs}^{h}\right)}_{dem,j}-{\left(E_{EDs}^{h}\right)}_{gen,j}).$$ (19)

$$min\sum_{h=1}^{H}P_{Dah}^{h}\times ({\left(E_{EDs}^{h}\right)}_{dem,j}-{\left(E_{EDs}^{h}\right)}_{gen,j}).$$ (20)

s.t.


$$0\leq {\left(E_{ED}^{h}\right)}_{gen,j}\leq {\left(E_{ED,r}\right)}_{gen,j},$$ (21)

$$0\leq {\left(E_{ED}^{h}\right)}_{dem,j}\leq {\left(E_{ED,max}\right)}_{dem,j},$$ (22)

$$0\leq {\left(E_{ED}\right)}_{c,j}\leq max(0,{\left(E_{EDs}^{h}\right)}_{dem,j}-{\left(E_{ED}^{h}\right)}_{gen,j}).$$ (23)

PEC of prosumers is in the range of minimum energy cost to maximum energy cost defined as:

$${\left(E_{ED}\right)}_{c,j}=\left\{ \begin{array}{c} {\left(E_{ED,min}\right)}_{c,j},\;for\;({\left(E_{ED,min}\right)}_{dem,j}\&{\left(E_{ED,max}^{h}\right)}_{gen,j})\\ {\left(E_{ED,mean}\right)}_{c,j},\;for\;({\left(E_{ED,mean}\right)}_{dem,j}\&{\left(E_{ED,mean}^{h}\right)}_{gen,j})\\ {\left(E_{ED,max}\right)}_{c,j},\;for\;({\left(E_{ED,max}\right)}_{dem,j}\&{\left(E_{ED,min}^{h}\right)}_{gen,j}) \end{array}\right.$$ (24)

where, $P_{RTP}^{h}$ and $P_{DaH}^{h}$ represents the RTP and DaH hourly pricing, (*E*_*ED*_)_*c*,*j*_ represents the actual energy cost of *j*^*th*^ ED while (*E*_*ED*,*min*_)_*c*,*j*_, (*E*_*ED*,*mean*_)_*c*,*j*_ and (*E*_*ED*,*max*_)_*c*,*j*_ represents the minimum, mean, and maximum energy cost of *j*^*th*^ ED respectively.

### 4.6 Grid revenue

The smart grid is facilitating the prosumer to increase their energy generation using renewable energy sources. The prosumer bidirectional energy flow between utility and prosumers is beneficial for all prosumers, utility, and the community. GR is maximized in the proposed SA-SLA based on RTP and DaH pricing schemes as decided by CM. The robust optimization problem formulation of the GR along with physical constraints is formulated as:

$$max\sum_{h=1}^{H}\left(P_{RTP}^{h}\times E_{u}^{h}+{\left(E_{ED}^{h}\right)}_{s,j}\times (P_{RTP}^{h}-P_{n}^{h})\right)$$ (25)

$$max\sum_{h=1}^{H}\left(P_{Dah}^{h}\times E_{u}^{h}+{\left(E_{ED}^{h}\right)}_{s,j}\times (P_{Dah}^{h}-P_{n}^{h})\right)$$ (26)

s.t.


$$0\leq {\left(E_{ED}^{h}\right)}_{gen,j}\leq {\left(E_{ED,r}\right)}_{gen,j},$$ (27)

$$0\leq {\left(E_{ED}^{h}\right)}_{dem,j}\leq {\left(E_{ED,max}\right)}_{dem,j},$$ (28)

$$0\leq {\left(E_{ED}\right)}_{s,j}\leq max(0,{\left(E_{ED}^{h}\right)}_{gen,j}-{\left(E_{ED}^{h}\right)}_{dem,j}),$$ (29)

$$0\leq E_{u}^{h}\leq max(0,{\left(E_{ED}^{h}\right)}_{dem,j}-{\left(E_{ED}^{h}\right)}_{gen,j}).$$ (30)

GR of the smart grid is in the range of minimum GR to maximum GR defined as:

$$GR=\left\{ \begin{array}{c} {GR}_{min},\;for\;(E_{u,min}^{h}\&P_{n,max}^{h})\\ {GR}_{mean},\;for\;(E_{u,mean}^{h}\&P_{n,mean}^{h})\\ {GR}_{max},\;for\;(E_{u,max}^{h}\&P_{n,min}^{h}) \end{array}\right.$$ (31)

where $P_{n}^{h}$ represents the nominal price for ED energy export, $E_{u}^{h}$ is the hourly energy import from utility while $E_{u,min}^{h},E_{u,mean}^{h}$, and $E_{u,max}^{h}$ denotes the hourly minimum, mean, and maximum energy import from utility respectively. $P_{n,max}^{h},P_{n,mean}^{h}$ and $P_{n,min}^{h}$ represents the hourly maximum, mean, and minimum nominal price rate respectively. *GR* is the actual GR of the smart grid while *GR*_*min*_, *GR*_*mean*_ and *GR*_*max*_ represents the minimum, mean, and maximum GR of the smart grid, respectively.

### 4.7 PES maximization using the simplex method

The PES of eDs is utilized to meet stochastic utility energy demand. The simplex method is used to maximize PES with stochastic utility energy satisfaction. Prosumer’s energy generation is considered daily in which each ED operates 24 hours of the day to meet stochastic energy demand. Each ED has gaussian *i*.*i*.*d* power generation and surplus $P_{s,1}^{h},P_{s,2}^{h},P_{s,3}^{h}$ with its capital and maintenance cost. *C*_*ED*1_, *C*_*ED*2_, and *C*_*ED*3_ are a total associated cost of ED1, ED2, and ED3 and $T_{1},T_{2},T_{3}$ denote the time of operation of ED1, ED2, and ED3 respectively. PES is maximized while minimizing PEC and this maximized PES is utilized to meet utility energy demand. The problem formulation is expressed as:

$$max\sum_{h=1}^{T_{1}+T_{2}+T_{3}}T_{1}P_{s,1}^{h}+T_{2}P_{s,2}^{h}+T_{3}P_{s,3}^{h}$$ (32)

*s*.*t*.

$$T_{1}C_{ED1}+T_{2}C_{ED2}+T_{3}C_{ED3}=C_{EDt}$$ (33)

$$T_{1}+T_{2}+T_{3}≼72,$$ (34)

$$T_{1},T_{1},T_{1}≽0.$$ (35)

where *C*_*EDt*_ is the total cost associated with all eDs.

### 4.8 Genetic Algorithm (GA) optimization

GA is an evolutionary algorithm for optimizing both constrained and unconstrained optimization problems. GA incorporates functions, namely objective function, fitness function, and genetic operators, such as mutation operator, crossover operator, and reproduction operator. The mutation is the flopping of randomly selected bits with a small mutation probability $p_{m}$ while the crossover is the main genetic operator usually associated with a higher probability $p_{c}$. Fitness functions are formulated as objective functions and represented as *F*_*S*_, *F*_*C*_, & *F*_*GR*_ for PES, PEC, and GR respectively. Genetic operators are applied to the old solutions and each new iteration, based on the old solution results in a new optimal point. The layout of a Genetic Algorithm for MTM is presented as:

**Algorithm 1** Genetic Optimization Algorithm for improved PES, PEC, and GR

1: **Initialize** with inputs ${\left(E_{ED}^{h}\right)}_{gen,j}$, ${\left(E_{ED}^{h}\right)}_{dem,j},P_{RTP}^{h}$, and $P_{Dah}^{h},P_{n}^{h}$.

2: **t =** 0.

3: **Evaluate *F***_***S***_**, *F***_***C***_, and *F*_*GR*_ using (14–31).

4: **If *F***_***S***_**, *F***_***C***_**, & *F***_***GR***_ converges, **then,**

5: **PES = (*E***_***ED***_**)**_***s*,*j***_, **PEC = (*E***_***ED***_**)**_***c*,*j***_, and **GR = *GR***.

6: **Else**

7: **t =** t+1

8: **Calculate** next generation individuals ${\left(E_{ED}^{h}\right)}_{gen,j}$, ${\left(E_{ED}^{h}\right)}_{dem,j},P_{RTP}^{h}$, and $P_{Dah}^{h},P_{n}^{h}$ using (15), (16), (25), and (26).

9: **Repeat** steps 3 and 4.

10: **End**.

### 4.9 Demand-supply management using the simplex method and ELD

In a smart grid, the energy balance state is maintained by mutual coordination of eDs prosumers and utility through bidirectional energy and information flow. For this purpose, the simplex method maximization and the problem of ELD are proposed for eDs that ensure a balance between utility energy demand and prosumer energy supply.

The simplex method maximizes the PES and selects the best possible combination (ratio) of the eDs operating time to meet utility energy demand with minimized eDs operating cost. The ELD calculates the eDs energy surplus with the corresponding eDs total operating cost. The objective is to determine whether PES is enough to meet utility energy demand with minimized eDs operating cost and extra energy (if available) after meeting the utility energy demand. The following are algorithms 2 and 3, which are presented for optimal utility energy demand management. The *SCs*-based SA-SLA algorithm is presented as:

**Algorithm 2** SA-SLA Algorithm to develop *SCs* based Smart SLAs

1: **Initialize** with inputs $WS,SI,{\left(E_{ED}^{h}\right)}_{dem,j},P_{RTP}^{h},\&P_{Dah}^{h},P_{n}^{h}$.

2: **Calculate** stochastically ${\left(E_{ED}^{h}\right)}_{gen,j}$ using (7–9).

3: **Compute (*E***_***ED***_**)**_***s*,*j***_ using (14).

4: **Select** corresponding *SC* using (1).

5: **Run** optimization algorithm,

6: **Compute** optimal PES, PEC, and GR using Algorithm 1.

7: **Run** Simplex Method,

8: **Maximize** (*P*_*EDs*_)_*s*_ to meet *P*_*U*_ using (32–35).

9: **Run** ELD,

10: **Find** (*P*_*EDs*_)_*s*_ = *P*_*U*_ using Algorithm 2.

11: **Develop** Smart SLAs **based** on *SC*, (*E*_*ED*_)_*s*,*j*_, & (*E*_*ED*_)_*c*,*j*_.

12: **End**

**Algorithm 3** ELD Algorithm to meet Utility Energy Demand

1: **Initialization** with inputs (*P*_*ED*_)_*s*,*j*,_ *P*_*U*,_ *C*_*EDj*_, and *Q*_*EDjj*_.

2: Stochastic (*P*_*EDs*_)_*s*_ is calculated using (48).

3: **If** (*P*_*EDs*_)_*s*_≥***P***_***U***_, ***then*,**

4: Compute *C*_*EDj*,*t*_ in (45),

5: Calculate (*P*_*EDs*_)_*s*,*new*_.

6: **If** (*P*_*EDs*_)_*s*,*new*_ = *P*_*U*_, ***then*,**

7: (*P*_*EDs*_)_*s*,*diff*_ = (*P*_*EDs*_)_*s*_−(*P*_*EDs*_)_*s*,*new*_.

8: **Else**

9: Repeat step 3.

10: **Else**

11: Calculate (*P*_*EDs*_)_*s*,*new*_.

12: **If** (*P*_*EDs*_)_*s*,*new*_ = *P*_*U*_, ***then*,**

13: (*P*_*EDs*_)_*s*,*diff*_ = (*P*_*EDs*_)_*s*,*new*_−(*P*_*EDs*_)_*s*_.

14: **Else**

15: Repeat step 12.

16: **End**

17: **End**

ELD is a technique to determine the optimal output power of multiple simultaneously running generating stations to satisfy stochastic energy demand with minimum cost. Fossil fuel-based generating stations are considered in ELD with several generating units in each station. In such types of generating stations, fuel is the major factor that contributes to the operating cost of generating stations. Fuel consumption varies in direct proportion to the power delivered by generating stations. Thus, to minimize the overall operating cost of several generating stations, it is demanding to optimally determine the output power of each generating station necessary to fulfill stochastic power demand.

For eDs with renewable energy generation facilities, the stochastic natural resources (wind speed, solar irradiance) are used as fuel. So, renewable-based eDs have only maintenance costs associated with them. Their operating (maintenance) cost remains the same irrespective of whether minimum or full rated power is supplied. Therefore, the output power reduction of renewable-based eDs may increase the fuel consumption leading to a high cost of generation. Therefore, there is an emergent need to model ELD for stochastic renewable-based eDs to (a) optimally determine output power for utility energy demand satisfaction and (b) the extra output power after demand fulfillment that can be utilized for any other purpose, such as storing in rechargeable batteries or supply to neighboring prosumer. So, eDs will avail extra advantage of utilizing this stored energy at the time of energy shortage. For a total of *n* different stochastic renewable-based eDs, the ED set N is defined as:

N=1,2,3,………,n. (36)

Each ED constitutes a unique stochastic power surplus *P*_*EDj*_. The total stochastic power surplus of eDs at a specified instant will be the sum of their stochastic output power. Total stochastic energy surplus eDs at a specified instant will be the sum of their stochastic output energies defined as:

$${\left(P_{ED}\right)}_{s}={\left(P_{ED}\right)}_{s1}+{\left(P_{ED}\right)}_{s2}+{\left(P_{ED}\right)}_{s3}+\cdots +{\left(P_{ED}\right)}_{sn}=\sum_{j=1}^{n}{\left(P_{ED}\right)}_{s,j}.$$ (37)

$${\left(E_{ED}\right)}_{s}={\left(E_{ED}\right)}_{s1}+{\left(E_{ED}\right)}_{s2}+{\left(E_{ED}\right)}_{s3}+\cdots +{\left(E_{ED}\right)}_{sn}=\sum_{j=1}^{n}{\left(E_{ED}\right)}_{s,j}.$$ (38)

where ${\left(E_{ED}\right)}_{s1},{\left(E_{ED}\right)}_{s2},{\left(E_{ED}\right)}_{s3},\ldots ,{\left(E_{ED}\right)}_{sn}$ are the energy surplus of eDs from 1 to *n* and depend on stochastic environmental parameters. The total operating (maintenance) cost of eDs is defined as:

$$C_{EDs}=C_{ED1}+C_{ED2}+C_{ED3}+\cdots +C_{EDn}.$$ (39)

$$C_{EDs}=\sum_{j=1}^{n}C_{EDj}.$$ (40)

where $C_{ED1},C_{ED2},C_{ED3},\ldots \ldots ..,C_{EDn}$ refers to operating costs of eDs from 1 to n corresponding to the power surplus of ${\left(P_{ED}\right)}_{s1},{\left(P_{ED}\right)}_{s2},{\left(P_{ED}\right)}_{s3},\ldots \ldots ..,{\left(P_{ED}\right)}_{sn}$ of n^th^ ED, respectively. The utility energy demand is sinusoidally distributed over the time horizon to reduce complexity and is defined as:

$$P_{U}=A\;\sin\left(\frac{2\times pi\times horizon}{24}\right).$$ (41)

where A is the mean utility energy demand. In this paper, three renewable-based eDs are considered for stochastic energy generation. The first two eDs use wind turbines for energy generation while the energy is produced by PV arrays in the third ED. The power surplus of eDs will between the minimum and maximum limits are expressed as:

$${{\left(P_{ED}\right)}_{s1,min}≼(P_{ED})}_{s1}≼{\left(P_{ED}\right)}_{s1,max},$$ (42)

$${{\left(P_{ED}\right)}_{s2,min}≼(P_{ED})}_{s2}≼{\left(P_{ED}\right)}_{s2,max},$$ (43)

$${{\left(P_{ED}\right)}_{s3,min}≼(P_{ED})}_{s3}≼{\left(P_{ED}\right)}_{s3,max}.$$ (44)

The overall cost function of the eDs is defined as:

$$C_{EDj,t}=Q_{ED,jj}{\left({P_{ED}}^{2}\right)}_{s,j}+C_{EDj}{\left(P_{ED}\right)}_{s,j}.$$ (45)

where, *Q*_*ED*,*jj*_ is the quadratic scaling factor of *j*^*th*^ ED. The problem is to find the portion of the power delivered by each ED that is necessary to meet utility energy demand with minimum eD operating costs. Optimization problem formulation of ELD for stochastic renewable-based eDs is formulated as:

$$min\sum_{j=1}^{n}C_{EDj,t}.$$ (46)

*s*.*t*.

$${\left(P_{ED}\right)}_{sj,min}≼{\left(P_{ED}\right)}_{sj}≼{\left(P_{ED}\right)}_{sj,max},$$ (47)

$${\left(P_{EDs}\right)}_{s}={\left(P_{ED}\right)}_{s,1}+{\left(P_{ED}\right)}_{s,2}+{\left(P_{ED}\right)}_{s,3},$$ (48)

$$P_{U}-{\left(P_{ED}\right)}_{s}≅0.0001,$$ (49)

$$P_{U}≽0,{\left(P_{ED}\right)}_{s}≽0.$$ (50)

where, *C*_*EDj*,*t*_ is the total cost of *j*^*th*^ ED while (PED)sj,min (*P*_*ED*_)_*sj*,*min*_ and (*P*_*ED*_)_*sj*,*max*_ are minimum and maximum PES of *j*^*th*^ eD. The Langrangian associated with *C*_*EDj*,*t*_ is defined as:

$$L(C_{EDj,t},\lambda ,\mu )=\sum_{j=1}^{n}C_{EDj,t}+\lambda h+\mu g.$$ (51)

where *L* represents Langrangian of total PES of all three eDs, h is inequality constraints of (*P*_*ED*_)_*s*_ used in (47) and (50), λ is the constant of multiplication for inequality constraints, g is the equality constraints of (*P*_*ED*_)_*s*_ and *P*_*U*_ used in (48) and (49), while *μ* is the constant of multiplication for equality constraints.

Stochastic environmental parameters (wind speed, solar irradiance) vary the PES of eDs between their minimum energy generation and maximum energy supply capacities. For PES, more than utility energy demand, the value of lambda is updated iteratively until the balance is achieved between PES and utility energy demand except for losses. The PES at this moment is called balanced PES. For PES, less than utility energy demand, the prosumer’s energy demand should decrease until the power balanced criterion satisfies defined as:

$${\left(P_{EDs}\right)}_{s}=P_{U}$$ (52)

## 5 Performance evaluation

### 5.1 Data analytics

Hourly data gathered from trustful sources [32] is considered for one year for three eDs located at Brownsville and Copano Bay Texas US. For day-wise simulations, the hourly available data is averaged for each day of the year. As the source parameters like wind speed and solar irradiance are stochastic and random, the considered data is almost aperiodic type. Seasonal data is also considered, such as winter, summer, and spring. Copano Bay has a Longitude of -97.0 and a Latitude of 28.1. Brownsville has Latitude and Longitude coordinated at 25.9 and -97.5 respectively. We considered WT installation at Copano Bay and solar PV panel installation at Brownsville due to important reasons like (a) having reasonable wind speed ranging from 3.7 m/s to 17.5 m/s enabling Copano Bay to produce enough energy generation and (b) Brownsville has significant solar irradiance to produce massive solar energy.

### 5.2 Seasonal variations

Prosumer’s energy consumption patterns and RES parameters show reliance on seasons of the year [33]. For better understanding, it is imperative to clarify the hitches of different seasons [34]. In this context, this paper presents the season-wise analysis of output parameters, such as PES, PEC, and GR. Moreover, a comparative analysis of both optimized and unoptimized results is provided.

#### 5.2.1 Spring season

**Fig 3:**
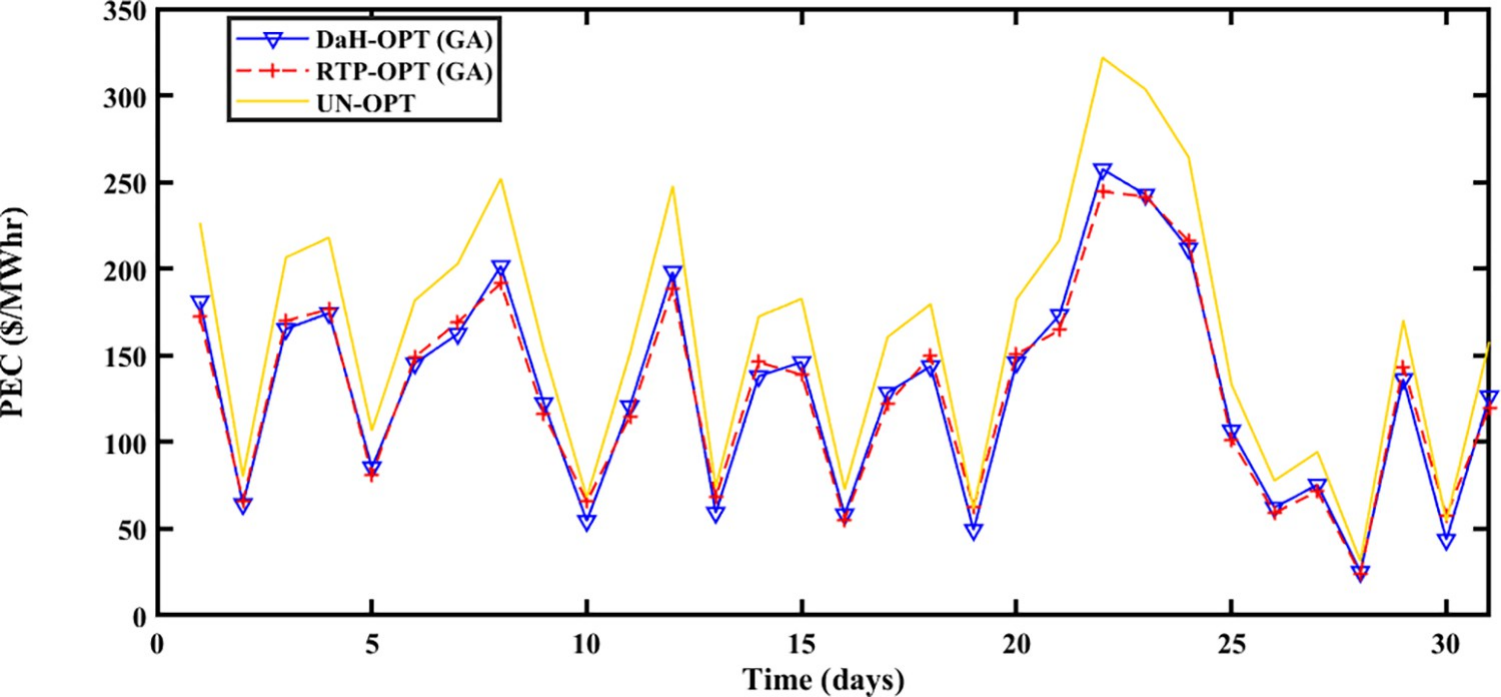
Prosumer energy cost of the spring season.

**Fig 4:**
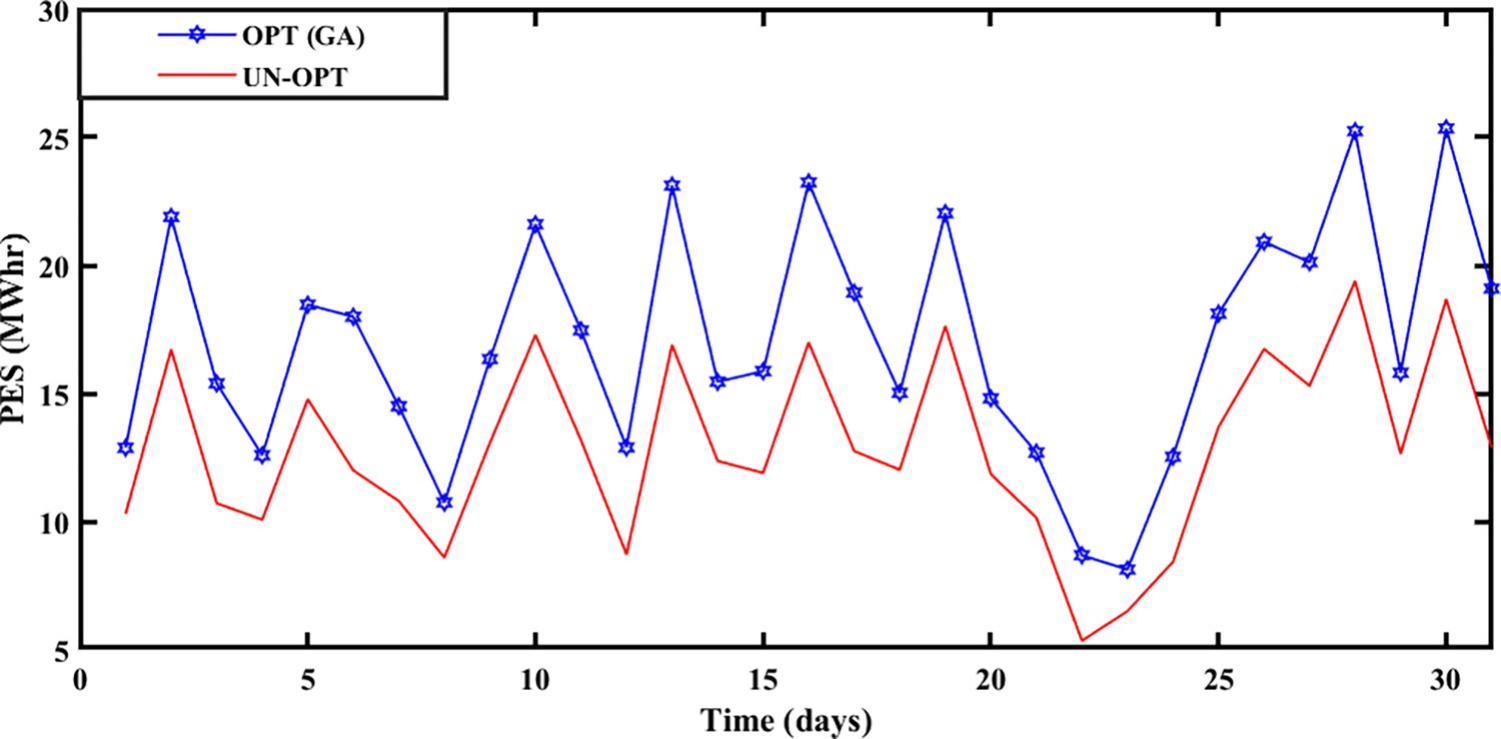
Prosumer energy surplus of the spring season.

**Fig 5:**
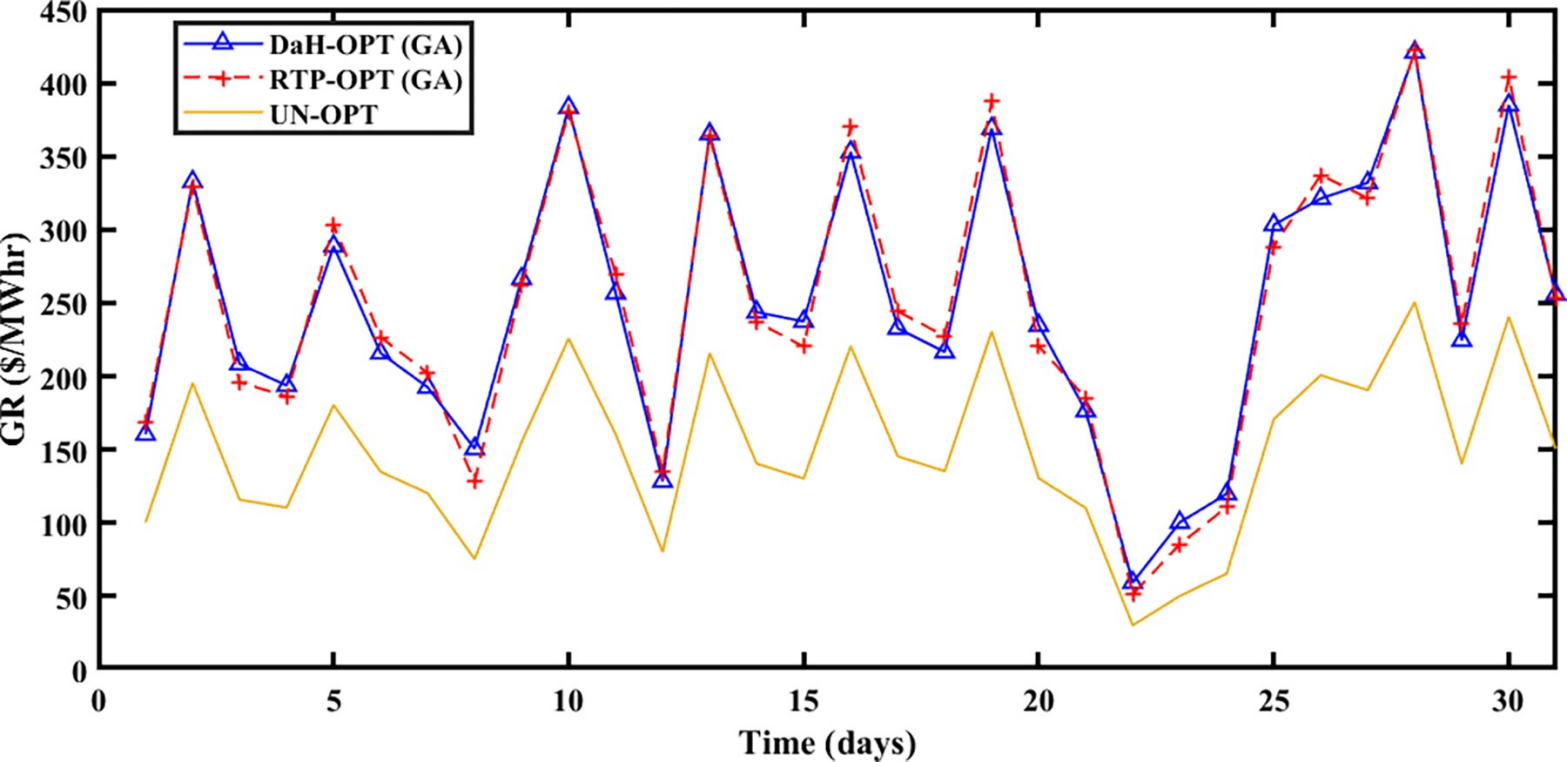
Grid revenue for the spring season.

The Spring season is observed during March, April, and May at Brownsville and Copano Bay Texas US. The month of March is selected from the spring season for analyzing the PEC, GR, and PES. During this season, a minimum number of heating and cooling devices are used, thus resulting in decreased energy demand with increased PES. The energy is imported and exported by prosumers under the umbrella of SA-SLA. Fig 3 shows the unoptimized (RTP) and optimized PEC. Fast convergence of SA-SLA is noticed on 22^nd^ March while convergence is slow on 28^th^ march. Fig 4 presents the unoptimized and optimized PES. Fig 4 shows that SA-SLA converges fast on 28^th^ march while the rate of convergence is slow on 22^nd^ March. The GR of the smart grid is presented in Fig 5 with a fast and slow convergence rate on the 28^th^ and 22^nd^ of March respectively.

#### 5.2.2 Summer season

**Fig 6:**
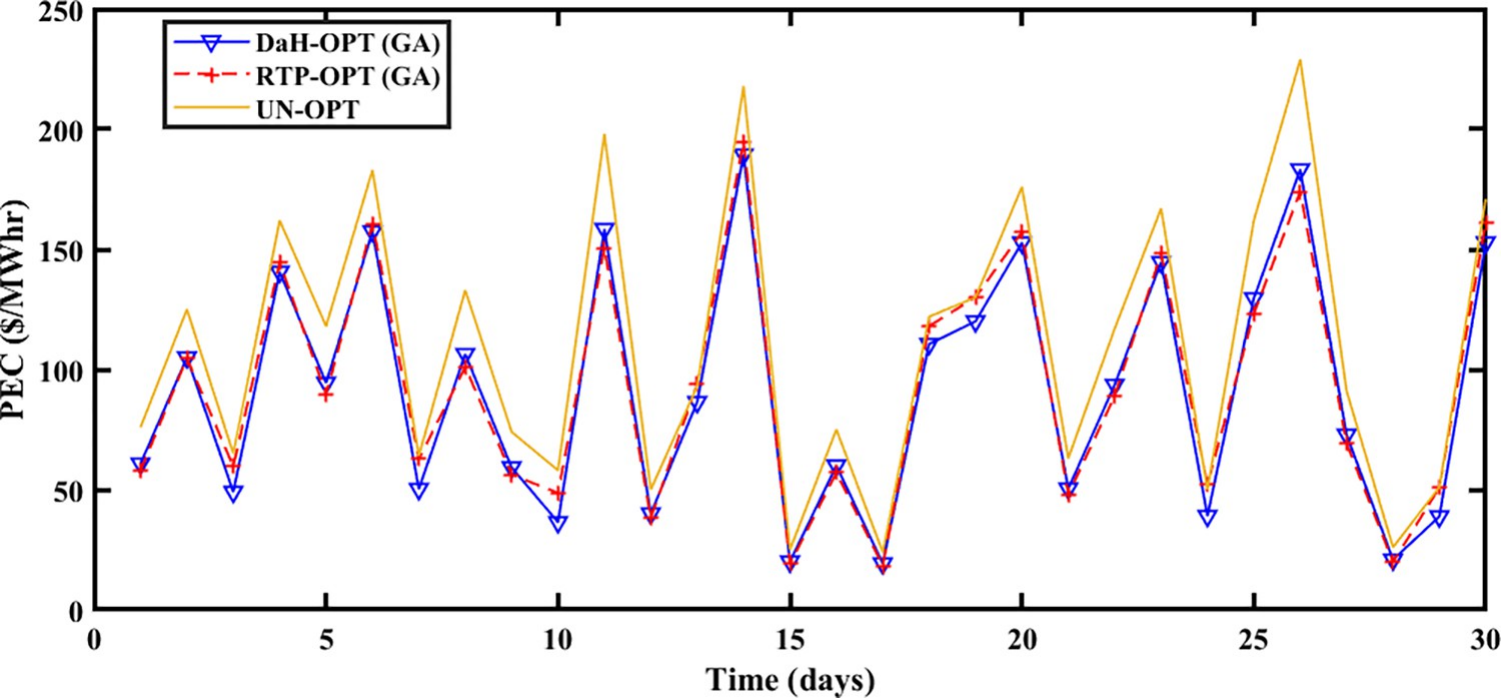
Prosumer energy cost of the summer season.

**Fig 7:**
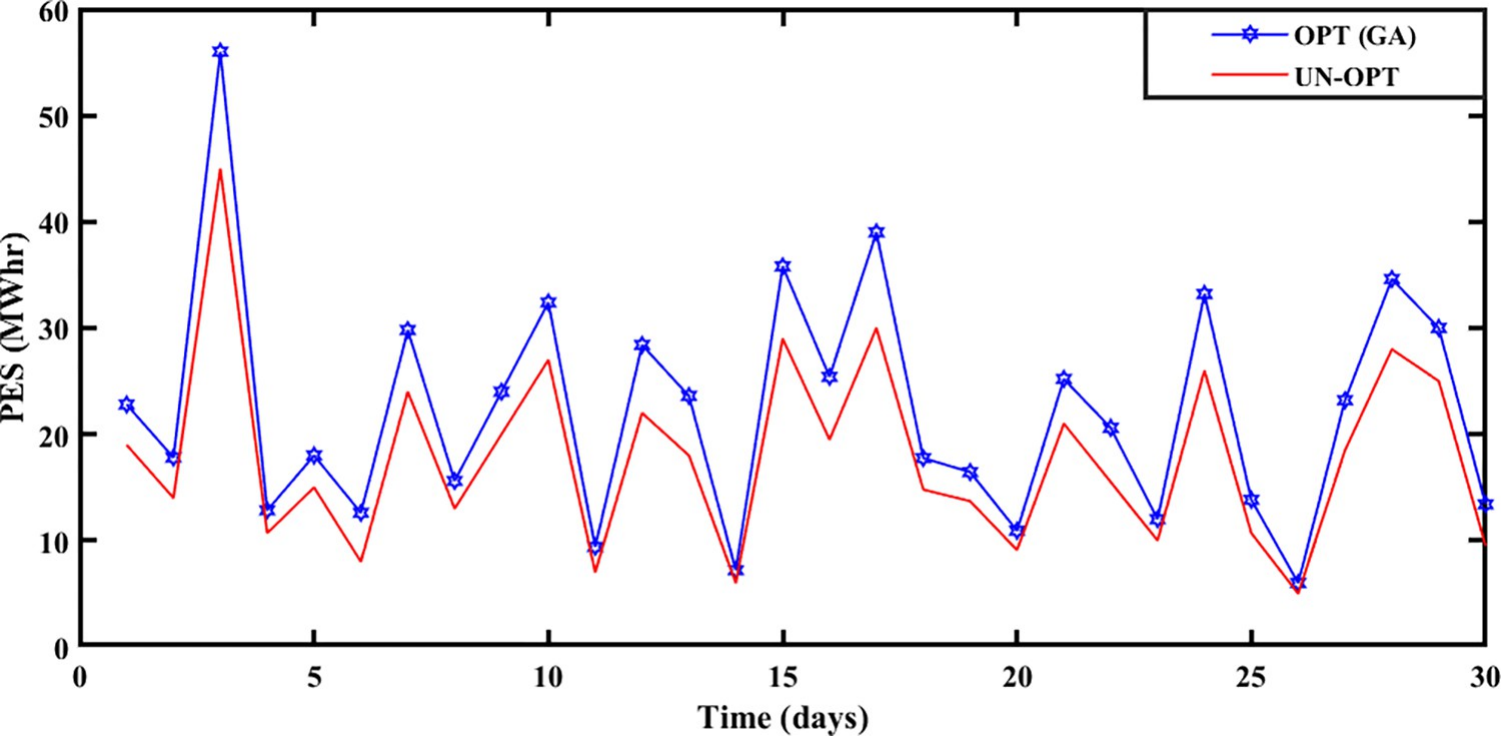
Prosumer energy surplus of the summer season.

**Fig 8:**
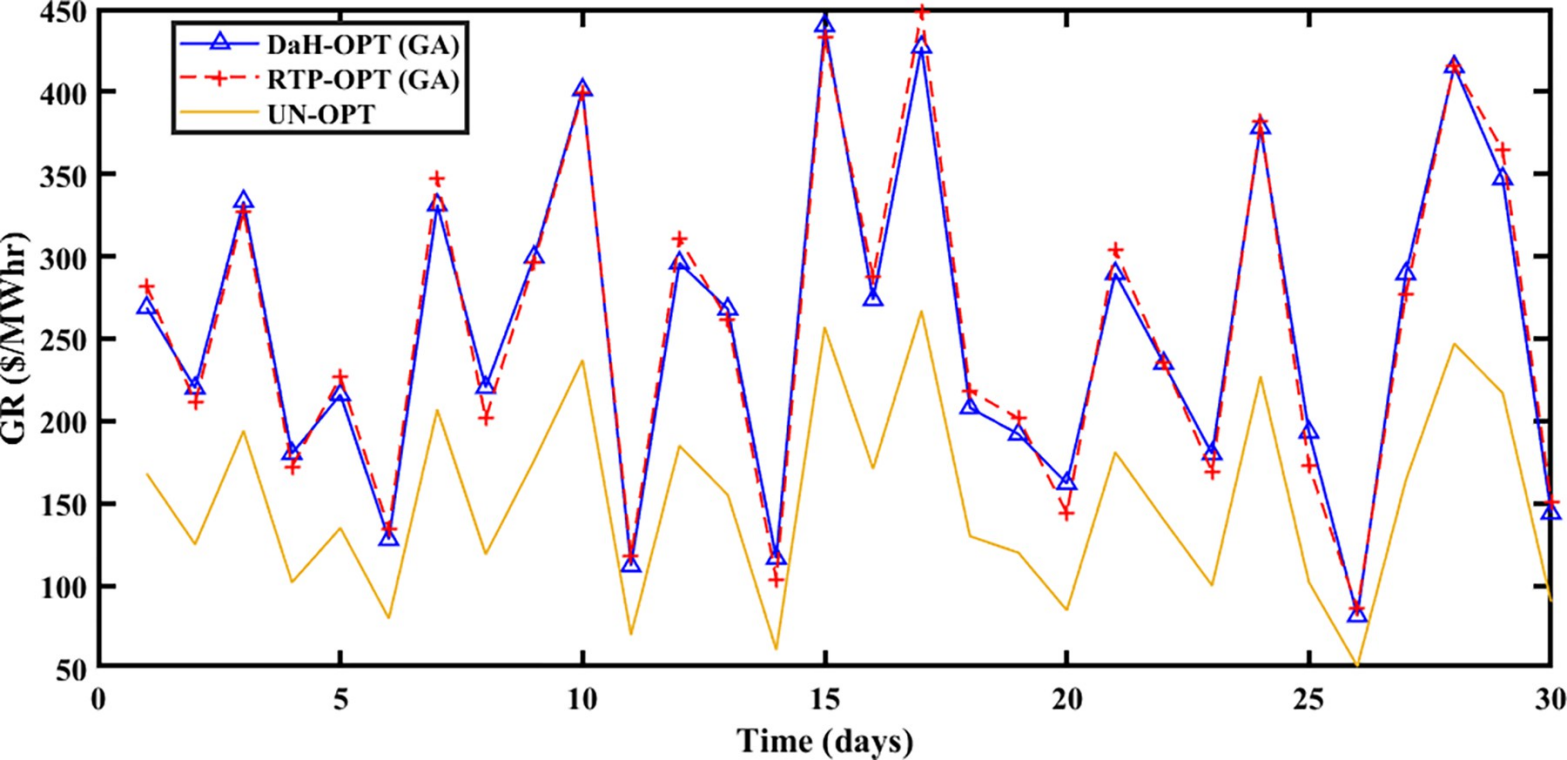
Grid revenue for the summer season.

The summer season starts from ‘June to September at Brownsville and Copano Bay Texas US. The month of June is selected from the summer season for analyzing the PEC, GR, and PES. In summer, maximum wind speed is observed resulting in the highest wind energy production in ED1 and ED2, compared to other seasons. Further, most summer days are sunny resulting in increased solar energy production in ED3. The hotness of summer compels prosumers to switch on the cooling devices for their comfort resulting in peak load, compared to other seasons. So, peak load is recorded in summer apart from maximum energy production resulting in maximum and minimum PEC and PES respectively. Fig 6 shows that the maximum PEC recorded on 26^th^ June and minimum PEC on 17^th^ June resulted in fast convergence of SA-SLA on 17^th^ June and slow convergence on 26^th^ June. Similarly, Fig 7 shows the fast convergence of SA-SLA on 3rd June and the slow convergence of SA-SLA on 26^th^ June for PES. The GR generated by SA-SLA is presented in Fig 8. The self-healing smart grid with the stochastic adaptive rate of convergence and minimum and maximum GR on 26^th^ and 15^th^ June results in fast and slow convergence respectively.

#### 5.2.3 Winter season

The Winter season starts in October and remains till the end of February at Brownsville and Copano Bay Texas US. The month of October is selected from the winter season for analyzing the PEC, GR, and PES. In the winter season, most of the days are cloudy and windy. So, reasonable wind energy with less solar energy generation (due to reduced solar irradiance) is possible. In the winter season, prosumer uses more heating devices, compared to other seasons resulting in peak load. Due to less solar energy production, the ED3 with PV panels imports more energy from the smart grid resulting in the highest PEC with slow SA-SLA convergence.

**Fig 9:**
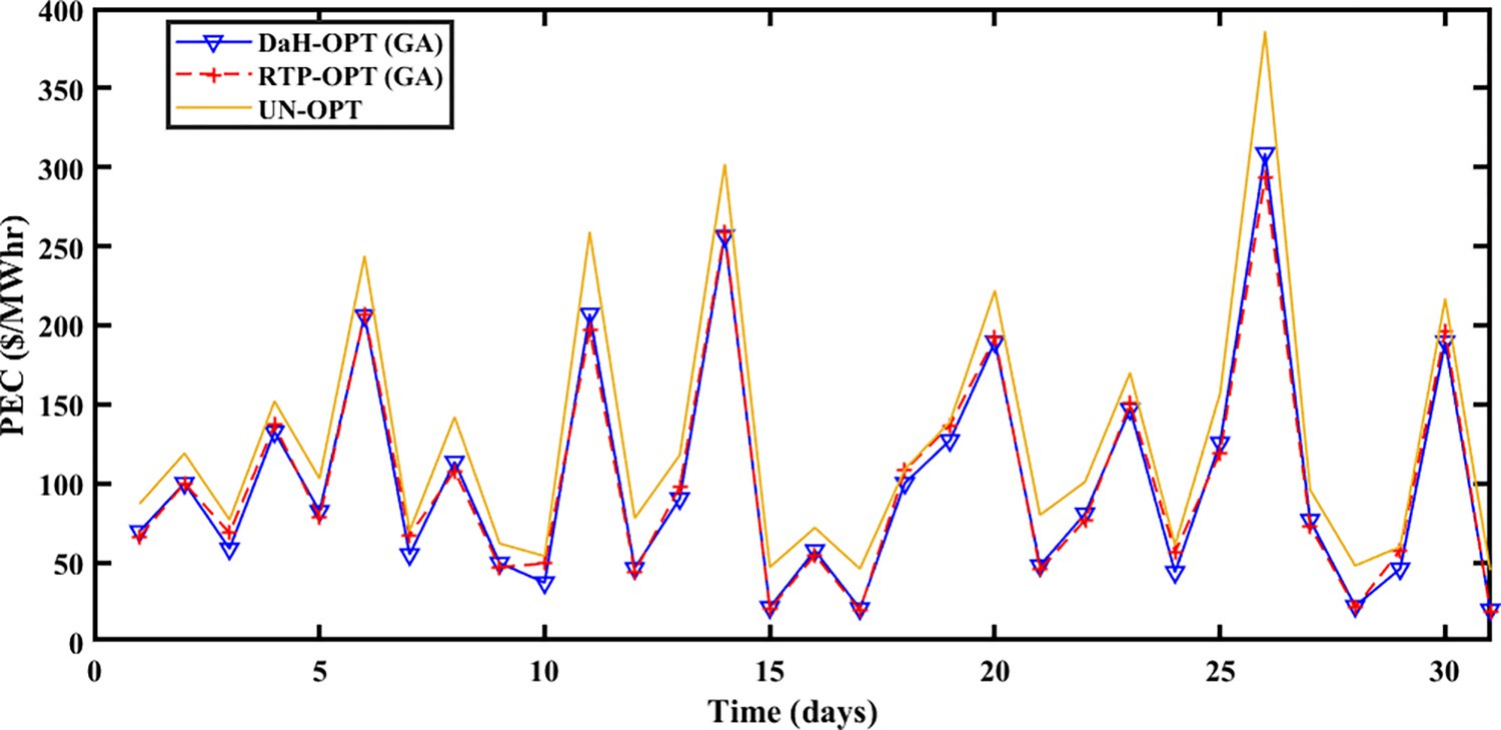
Prosumer energy cost of the winter season.

**Fig 10:**
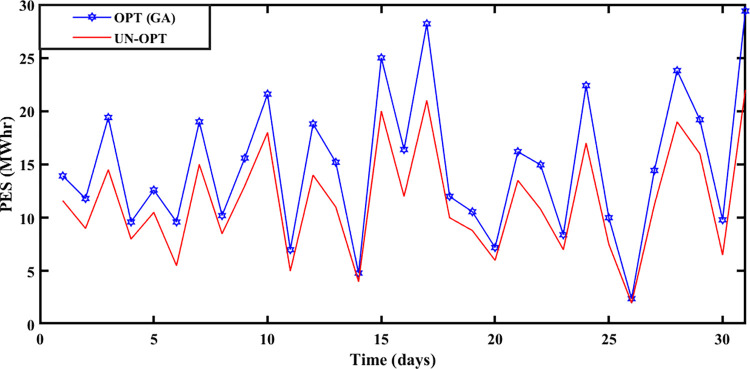
Prosumer energy surplus of the winter season.

**Fig 11:**
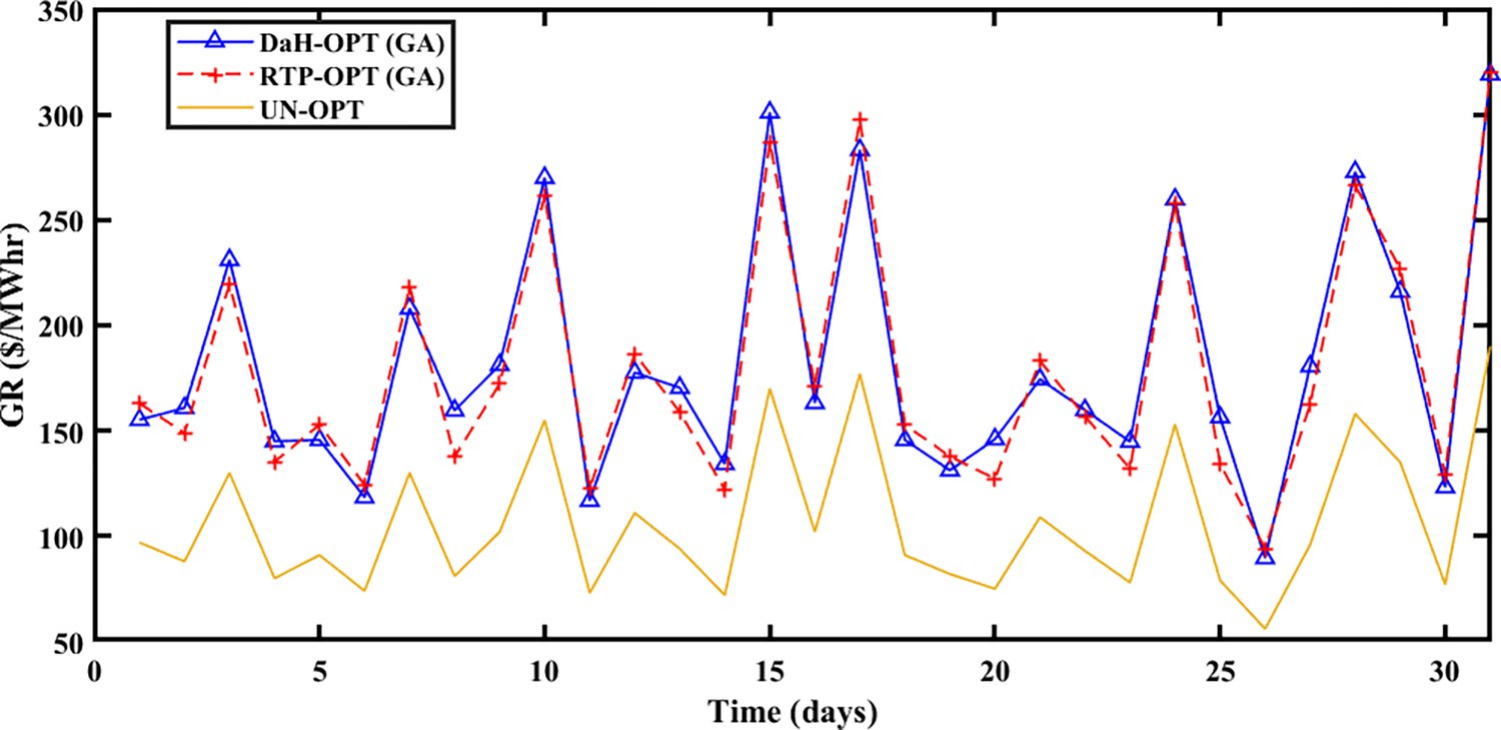
Grid revenue for the winter season.

PEC of prosumers is shown in Fig 9 with fast and slow SA-SLA convergence on 31^st^ and 26^th^ October respectively while PES is depicted in Fig 10 with fast and slow SA-SLA convergence on 26^th^ and 31^st^ October respectively. Fig 11 presents the GR of SG generated under SA-SLA by the virtue of stochastic adaptive SCs between stakeholders. Fast and slow SA-SLA convergence of GR is noticed on 26^th^ and 31^st^ October respectively.

### 5.3 Demand-supply optimized management

**Fig 12:**
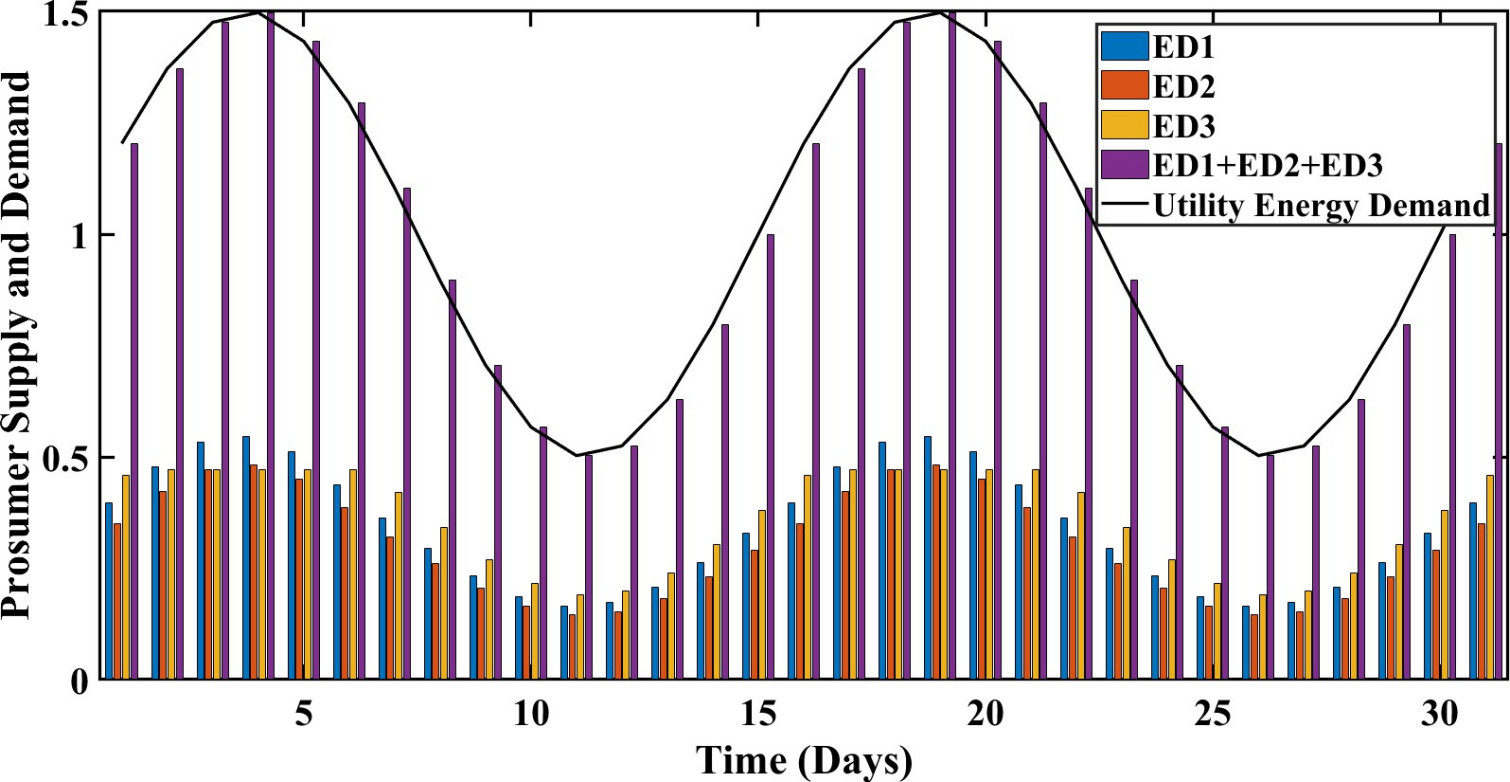
Demand-supply optimized management using the simplex method.

**Fig 13:**
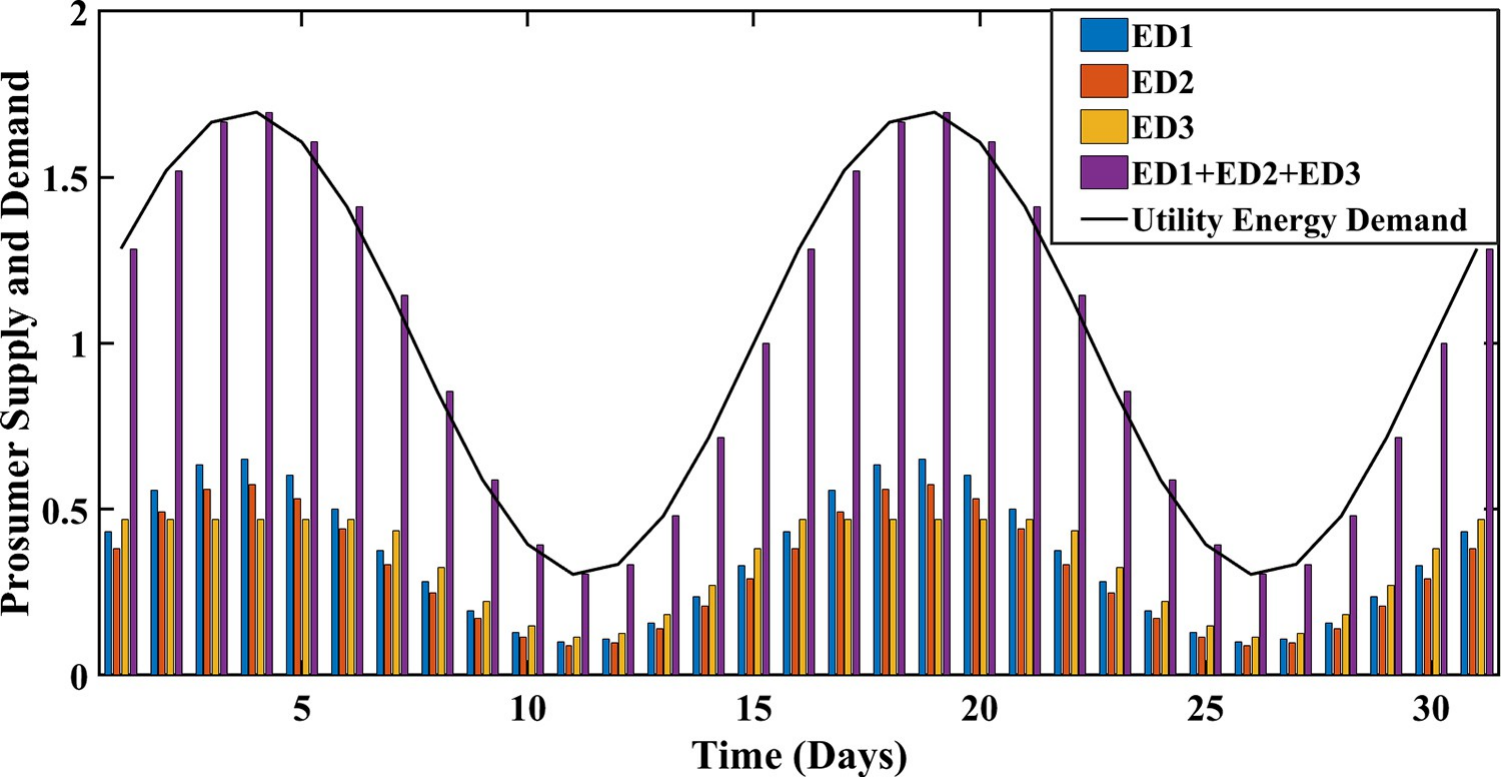
Demand-supply optimized management using ELD.

The proposed SA-SLA using the simplex method and ELD, guarantee a balance between energy supply and energy demand. The matched energy supply and energy demand increase the convergence rate of SA-SLA and GR of the smart grid. The simplex method and ELD are the two prominent techniques employed to concisely manage the energies. Utility energy demand and PES management using the simplex method and ELD are shown in Figs 12 and 13 respectively. The percentage increase in PES using ELD is 11.76 percent, compared to the simplex method. The balance between energy demand and energy supply is achieved with the advantage of extra surplus energy that can be stored or utilized for any other purpose. The comparison shows that the ELD technique outperforms the simplex method in terms of demand-supply optimized management.

### 5.4 Multiple SLAs convergence and divergence

The variations in stochastic wind speed and solar irradiance bring changes in PES and PEC of prosumers. PEC Susceptible RoC and RoD of SA-SLA are influenced by stochastically changing PES and PEC. Stochastic SCs are developed between stakeholders and based on these SCs, multiple SLAs are developed under SA-SLA. Each newly developed SLA comprises unique RoC and RoD. Convergence provides a sense of problem-solving (region of benefit) while divergence provides a sense of moving away from problem solution (region of benefit). The convergence and divergence of each newly developed SLA are based on the area under the PDF of gaussian i.i.d values of PES and PEC for specific SC. The centered 95% area under the PDF curve is considered the RoC of SA-SLA while the remaining 5% outer area is the RoD SA-SLA.

### 5.5 Multiple smart SLAs

The formal contract between the customer (eDs) and service provider (smart grid) with a set of defined service standards is called SLA. PES and PEC are dependent variables of renewable energy generation (wind and solar). The specific range of PES and PEC is contracted between stakeholders defining the RoC and RoD of a specific SLA. Fixed SLA associate static PES and PEC in the contract. The stochastic source parameters result in different PES, PEC, and GR of a smart grid that may undergo in RoD of fixed SLA and lead toward SLA violation. Thus, there was an emergent need to develop an SLA that increases stakeholders’ mutual benefits by incorporating a stochastic pattern of source parameters (wind speed and solar irradiance).

**Fig 14:**
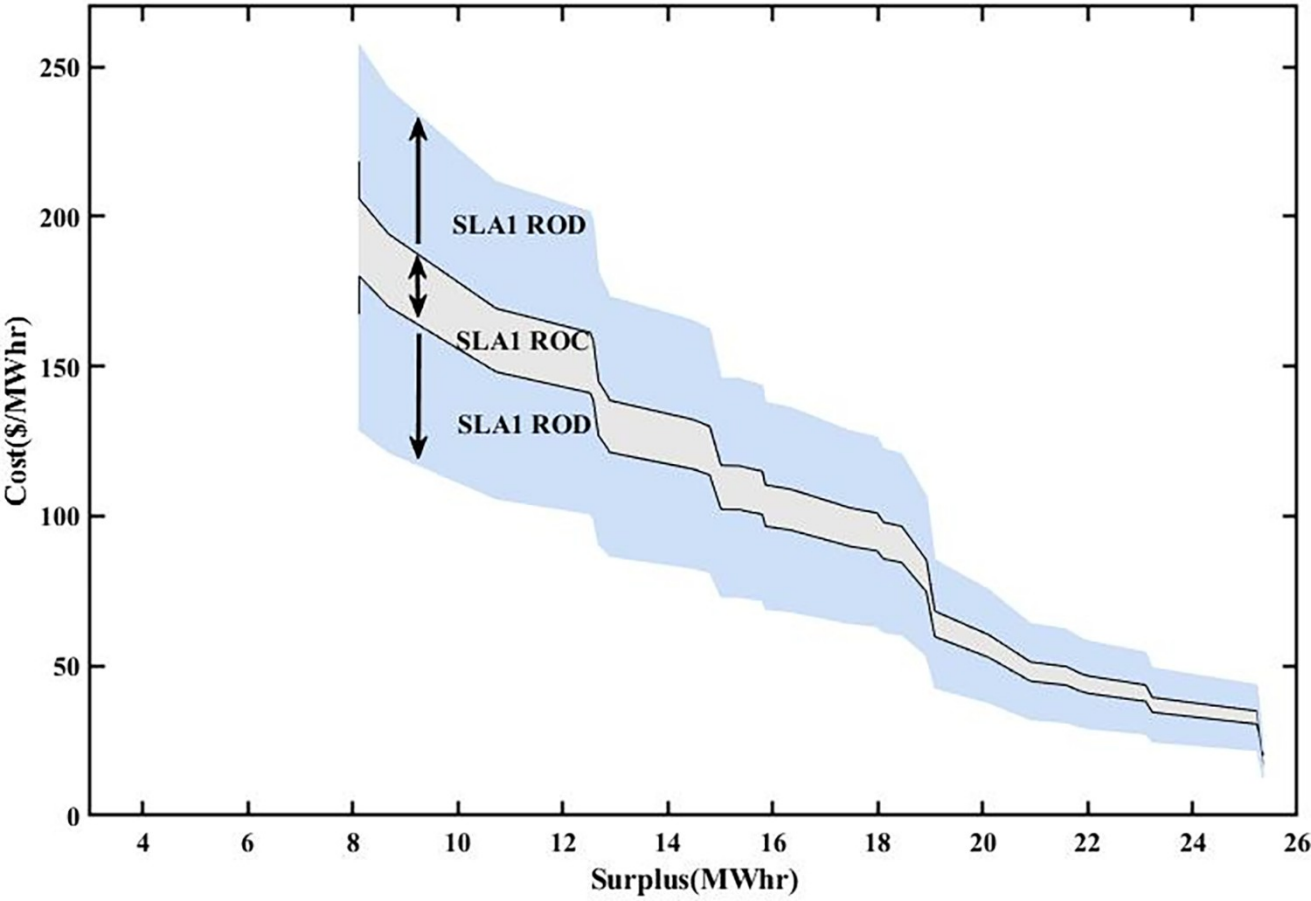
RoC and RoD of SLA1.

**Fig 15:**
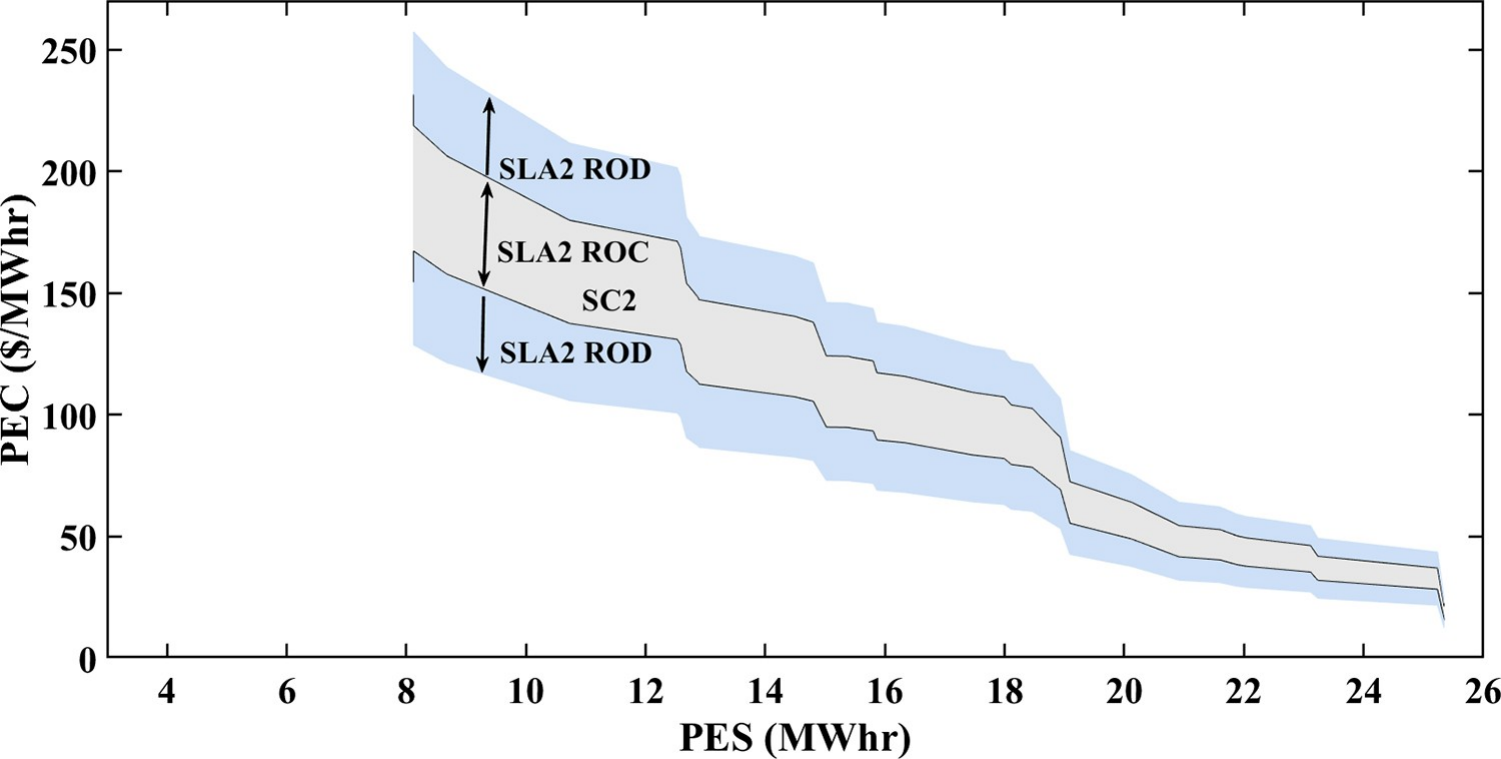
RoC and RoD of SLA2.

**Fig 16:**
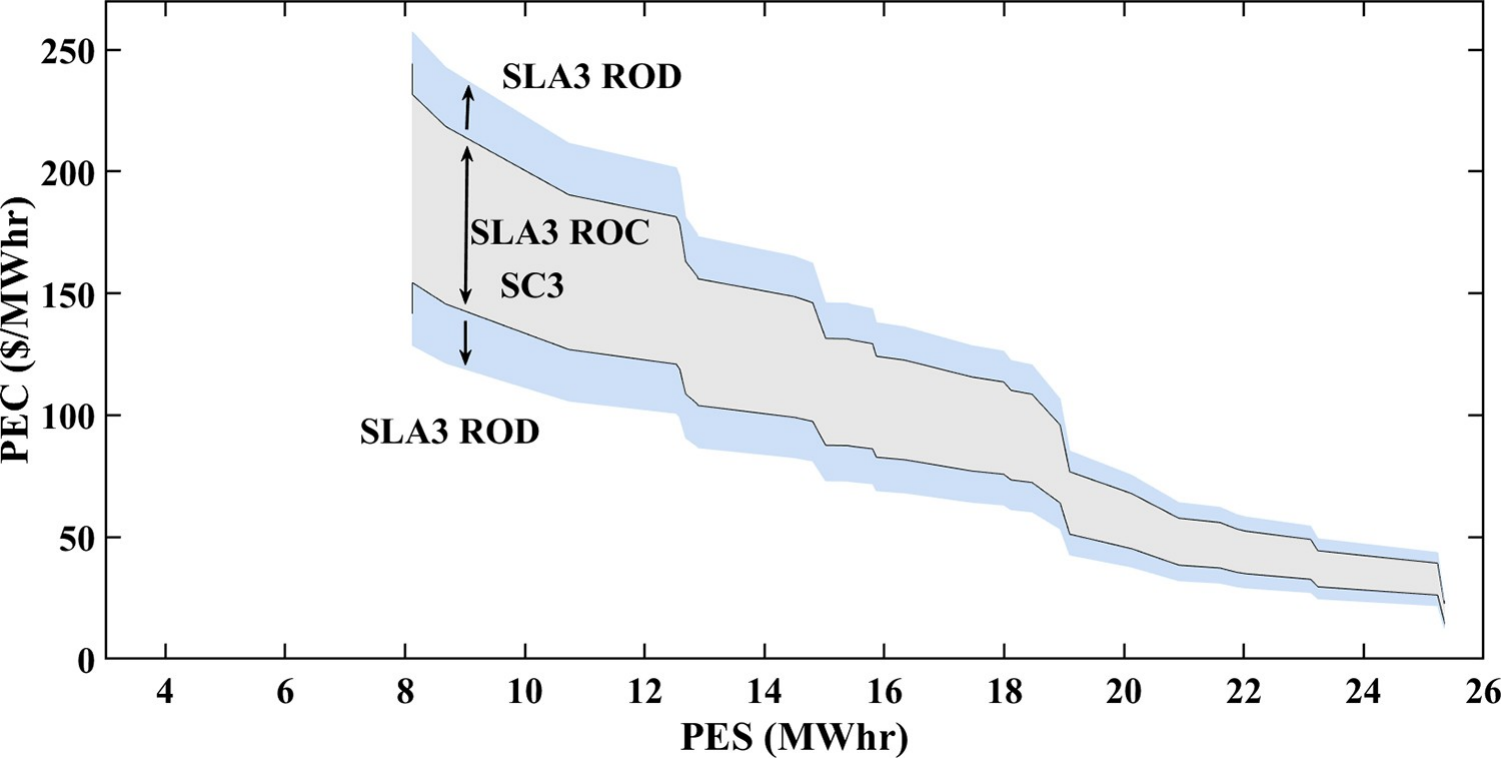
RoC and RoD of SLA3.

An SA-SLA is comprised of multiple smart contracts and SLAs are modeled and designed. Each time source parameters vary their dynamics, SCs-based new SLAs are developed and create multiple RoC and RoD of SA-SLA. Each newly developed SLA results in a unique RoC and RoD. The SA-SLA converges in the RoC region of a specific SLA and increases the mutual benefits of stakeholders, while diverges in the RoD region of a specific SLA, which is not beneficial to stakeholders. If SLA1 moves towards the divergence region due to unbounded values of PES and PEC at a specific hour or day, the possibility of convergence (converged values of PES and PEC) with any other contracted SLA is checked and adopted, having optimum values of PES and PEC at that specific moment. The convergence and divergence responses to multiple smart SLAs of SA-SLA are based on the area under the PDF of Gaussian distributed PES and PEC. For paradigm, three different SLAs developed are shown in Figs 14–16. At any instant of time with specific PES and PEC, the suitability of multiple smart SLAs is checked and validated for increased mutual benefits. SLA1, SLA2, and SLA3 have some common RoC, but there exists another region where SLA1 diverges while SLA2 shows convergence, as shown in Figs 14 and 15.

Similarly, Figs 15 and 16 depict that for the same region, the SLA2 divergences while SLA3 shows convergences. The proposed SA-SLA shows that the RoD region of SLA1 becomes the RoC of SLA2 and similarly, the RoD region of SLA2 becomes the RoC of SLA3. The RoC and RoD of multiple SLAs may completely interchange or overlap and must possess associated PES and PEC.

### 5.6 Statistical analysis

**Fig 17:**
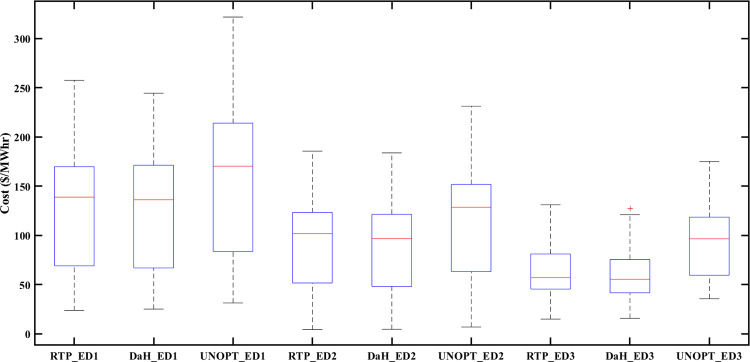
Box plot of PEC of eDs for comparison between optimized and unoptimized PEC. UNOPT: Un-Optimized.

Box-and-whisker plot (also known as a box plot) plays an important role in describing statistical characteristics of numeric data and is especially useful when multiple data sets are being compared. Visual representation of the box plot develops a sense of five important data characteristics, such as lowest observation, highest observation, lower quartile, upper quartile, and median. Lines outside the box are known as whiskers. Bars at the upper and lower tip of whiskers represent the highest and lowest observations while the median is represented by the line inside a box. Box plot analysis of PEC is provided in Fig 17. The vertical axis denotes prosumers cost (in $/Mwh) while the horizontal axis represents RTP optimized, DaH optimized, and unoptimized categories of prosumer cost for ED1, ED2, and ED3. Fig 17 depicts that optimized results of PEC are improved for all eDs. Moreover, it is noticeable that DaH optimization results of PEC are decreased in comparison with RTP, for all eDs.

**Fig 18:**
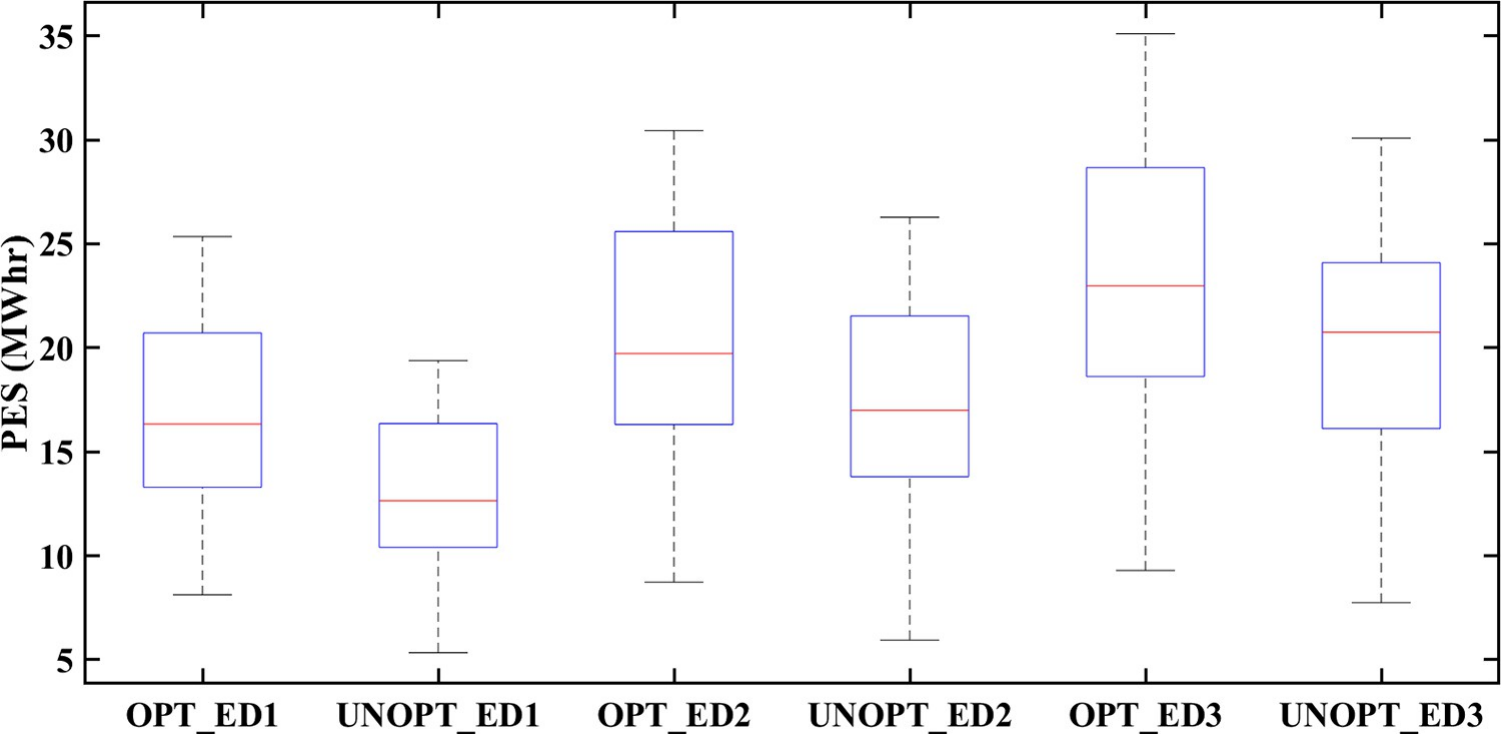
Box plot of PES of eDs for comparison between optimized and unoptimized PES.

**Fig 19:**
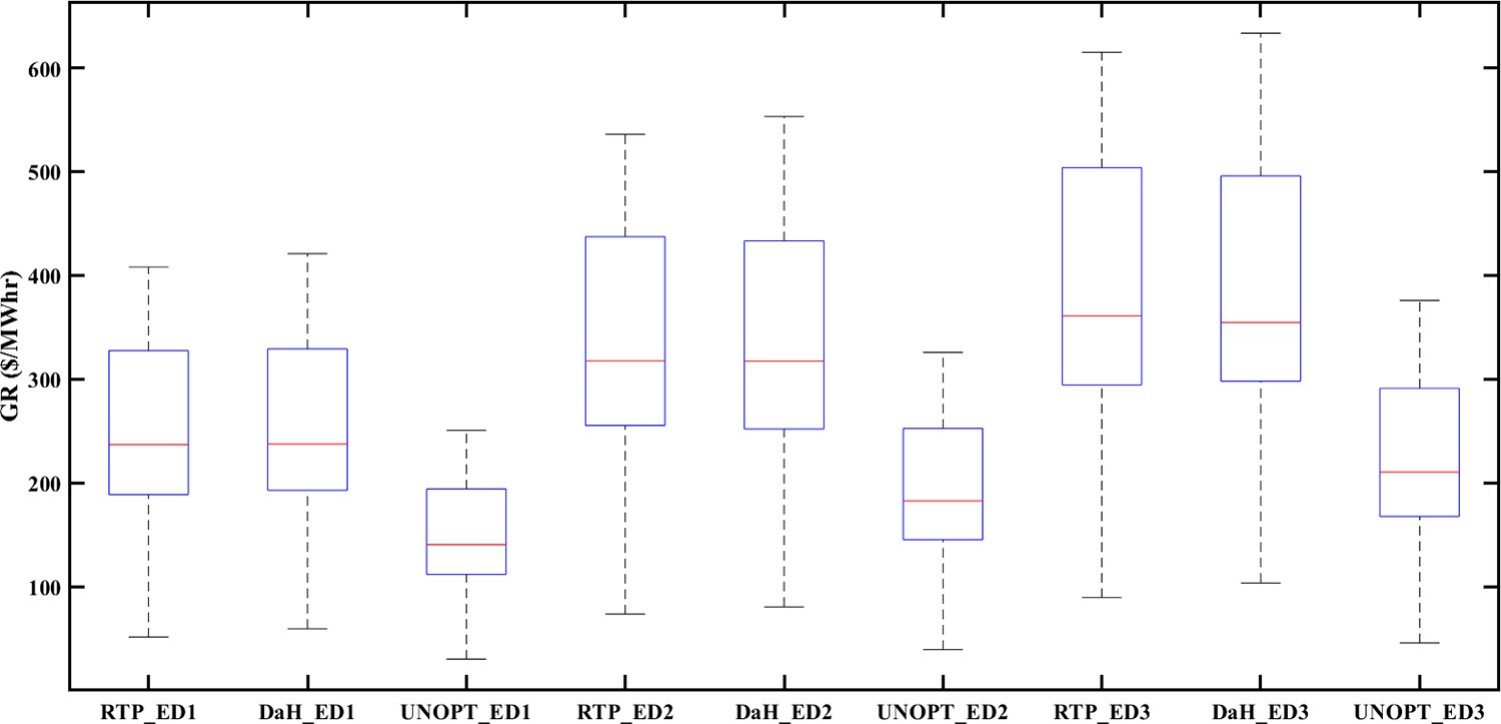
Box plot of GR of eDs for comparison between optimized and unoptimized GR.

Similarly, the box plot analysis of PES for all eDs is provided in Fig 18. The vertical axis of Fig 18 represents energy surplus (in Mwh) while the horizontal axis is labeled with optimized and unoptimized categories of prosumer energy surplus for ED1, ED2, and ED3. Fig 18 shows that optimized results of PES are better for all eDs. Prosumers’ participation in the smart grid, not only benefits prosumers but also increase the GR of the smart grid. Fig 19 represents a box plot analysis of GR, similarly to PEC with the only difference that instead of PEC, we consider GR (in $/Mwh) at the vertical axis of Fig 19. Improved results in terms of GR of smart grid with RTP-based optimization and DaH pricing-based optimization can be noticed in Fig 19. Moreover, Fig 19 also reveals that optimization based on DaH pricing generates more GR for the smart grid than optimization based on RTP. Therefore, it is concluded that increased mutual benefits of eDs prosumer and smart grid are associated with DaH pricing.

### 5.7 Tabular analysis

As explained earlier, both RoC and RoD are associated with SA-SLA. It is imperative to develop strong knowledge about the contractual limits of RoC and RoD as they decide whether bidirectional energy flow between stakeholders is beneficial or not. PEC, PES, and GR must remain in the corresponding convergence region for the mutual benefit of stakeholders. RoC and RoD of PES, GR, and PEC for ED1, ED2, and ED3 are tabulated in Table 1 for one month of each season (Mar for spring, June for summer, and October for winter) while an SC-based RoC for multiple smart SLAs of SA-SLA is tabulated in Table 2. From Tables 1 and 2, it is concluded that there are different convergence ranges for different SCs in terms of PEC and PES.

**Table 1:** Region of Convergence (RoC) and Region of Divergence (RoD) of PEC, PES, and grid revenue.

eDs	Months	RoC of PEC	RoD of PEC	RoC of PES	RoD of PES	RoC of GR	RoD of GR
**ED1**	Mar	321–118	321<C<118	25–1	25<Es<1	410–25	410<GR<25
	June	229–150	229<C<150	57–5	57<Es<5	450–50	450<GR<50
	Oct	386–126	386<C<126	29–3	29<Es<3	320–55	320<GR<55
**ED2**	Mar	225–170	225<C<170	30–6	30<Es<6	570–51	570<GR<51
	June	155–120	155<C<120	68–8	68<Es<8	600–90	600<GR<90
	Oct	271–156	271<C<156	37–3	37<Es<3	420–71	420<GR<71
**ED3**	Mar	172–123	172<C<123	35–8	35<Es<8	645–52	645<GR<52
	June	110–79	110<C<79	78–9	78<Es<9	674–96	674<GR<96
	Oct	207–146	207<C<146	42–3	42<Es<3	491–97	491<GR<97

**Table 2:** SCs-based RoC for multiple smart SLAs of SA-SLA.

Parameters	SLA1	SLA2	SLA3
PEC ($/Mwh)	218–24	231–23	248–22
PES (Mwh)	9–25.5	9–25.5	9–25.5

### 5.8 Critical debate

Output power from WT and solar PV array is the function of the stochastic source parameters (wind speed and solar irradiance) and is influenced by the stochasticity of source parameters. In this context, prosumers’ generated energy varies at every instant following the current statistic of stochastic source parameters. Variations encountered in prosumers generated energy stochastically change prosumers’ energy import and export and result in randomly distributing PES and PEC. Prosumer’s energy import and export activities are monitored by CM under the authority of SLA. Fixed SLA is based on a fixed contract between stakeholders and inflexible to the stochasticity of source paraments. In real-time scenarios, large variations are encountered in PES and because of fixed contract-based SLA, most PES occur outside the contractual limits. The purpose of utilizing almost all PES for utility energy management arises a need to design an SLA that may vary contractual limits for mutual benefits of stakeholders depending on available PES. A multiple SCs-based SA-SLA is one of the solutions to overcome the limitations of fixed SLA-based EMMs. SAFs of prosumers generated energy is modeled in SA-SLA that generate gaussian i.i.d values of prosumers generated energy. Gaussian i.i.d values of prosumers generated energy stochastically change PES. As PES can vary from minimum prosumer energy generation to their maximum energy generating capacities, various ranges of PES are defined in SC. As stochastic source parameters change their values, the new SCs are developed in terms of PES as defined in (1).

SC1 is responsible to develop SLA1 with high PEC and a small area of convergence as shown in Fig 14. This is because of the reason that minimum PES is associated with SC1. SC2 is liable to develop SLA2 with less PEC and a large area of convergence than SLA1 as shown in Fig 15. The PEC is mostly associated with the bottom portion of the convergence area revealing that the large PES of SC2 decreases PEC to some extent. Similarly, SC3 is liable to develop SLA3 with less PEC and a large area of convergence than SLA2 as shown in Fig 16. As the largest PES is associated with SC3, SLA3 has a wider convergence area. As moving further toward large PES in SLA1, SLA2, and SLA3, the PEC decreases but the difference is: that for the same PES, less PEC is associated with SLA2 and SLA3 than with SLA1 because of their contract.

Figs 14–16 that 95% convergence area under the PDF curve of PES associates more PES as the move from SLA1 to SLA3 and 95% convergence area under the PDF curve of PEC associates less PES as the move from SLA1 to SLA3. Table 2 shows the improved statistics of SA-SLA in terms of PES and PEC. The demand-supply optimized management is another important perspective of SA-SLA. As explained earlier, the stochastic parameters change PES in a stochastic manner. The variation in PES disturbs the energy balance of prosumers and utility. In response to stochastic PES, an optimized demand-supply management mechanism using the simplex method and ELD is developed and validated as clear in Figs 12 and 13. Moreover, ED1, ED2, and ED3 cumulatively put a great energy share to reduce the peak energy demand of the smart grid. The purpose of the above discussion is clear that SA-SLA with multiple SCs promotes prosumer participation in smart grid and increases the mutual benefits of stakeholders by developing multiple SLAs.

## 6 Conclusions

The penetration of renewable energy generation-based eDs into smart grids transforms the behavior of energy importing consumers (utilities) into active energy managing prosumers. The MTM between eDs and smart grid with multiple SCs increases the gain of both parties and flourishes the structure of the power system. The overall performance of the system is influenced by stochastic parameters and the role played by players, actors, and aggregators. In this paper, the stochastic and adaptive functions of prosumer-generated energy are incorporated due to the stochasticity of source parameters. Further, the new SLAs technique is provided to incorporate multiple ranges of PES. The weather’s parametric effects on prosumer energy consumption are modeled. The performance of the purposed SA-SLA is evaluated via a season-wise analysis of the year, namely the summer season, spring season, and winter season. The summer season accompanied by regular wind blowing and clear solar irradiance results in massive energy generation from RES. The analysis of the result shows a maximum PEC reduction (31%) in summer, compared to spring (28%) and winter (25%). Similarly, the PES maximization is recorded at 47% in summer than in spring (40%) and winter (41%). Moreover, the demand-supply optimized management achieved by ELD meets 11.76% more utility energy demand than the simplex method. Multiple contractual limits of smart SLAs result in decreased PEC with increased PES. Thus, multiple SCs-based SA-SLA, by developing multiple SLAs, increases the mutual benefits of stakeholders in terms of PEC, PES, and GR.

## Supporting information

S1 File (RAR)

## Nomenclature

CM: Coalition Manager
DaH: Day-a-Head PES
DSM: Demand Side Management
DRPs: Demand Response Programs
ELD: Economic Load Dispatch
eDs: Energy Districts
GR: Grid Revenue
i.i.d: Independent and Identically Distributed
MTM: Mutual Trade Model
PEC: Prosumer Energy Cost
PV: Photovoltaic
PDF: Probability Density Function
ROM: Robust Optimization Model
RoD: Region of Divergence
RoC: Region of Convergence
RTP: Real-Time Price
SAF: Stochastic Adaptive Function
SCs: Smart Contracts
SA-SLA: Stochastic Adaptive- Service Level Agreement
